# SEPN1-related myopathy depends on the oxidoreductase ERO1A and is druggable with the chemical chaperone TUDCA

**DOI:** 10.1016/j.xcrm.2024.101439

**Published:** 2024-02-22

**Authors:** Serena Germani, Andrew Tri Van Ho, Alessandro Cherubini, Ersilia Varone, Alexander Chernorudskiy, Giorgia Maria Renna, Stefano Fumagalli, Marco Gobbi, Jacopo Lucchetti, Marco Bolis, Luca Guarrera, Ilaria Craparotta, Giorgia Rastelli, Giorgia Piccoli, Cosimo de Napoli, Leonardo Nogara, Elena Poggio, Marisa Brini, Angela Cattaneo, Angela Bachi, Thomas Simmen, Tito Calì, Susana Quijano-Roy, Simona Boncompagni, Bert Blaauw, Ana Ferreiro, Ester Zito

**Affiliations:** 1 Istituto di Ricerche Farmacologiche Mario Negri IRCCS, Milan, Italy; 2 Department of Biomolecular Sciences, University of Urbino Carlo Bo, Urbino, Italy; 3 Department of Molecular and Developmental Medicine, University of Siena, Siena, Italy; 4 Basic and Translational Myology Laboratory, Université Paris, BFA, UMR 8251, CNRS, 75013 Paris, France; 5 Bioinformatics Core Unit, Institute of Oncology Research (IOR), 6500 Bellinzona, Switzerland; 6 CAST, Center for Advanced Studies and Technology & DNICS, Department of Neuroscience, Imaging and Clinical Sciences, University G. D’Annunzio of Chieti-Pescara, 66100 Chieti, Italy; 7 Department of Biomedical Sciences, University of Padua, Padua, Italy; 8 Department of Pharmaceutical Sciences, University of Padova, Padova, Italy; 9 Department of Biology, University of Padova, Padova, Italy; 10 Study Center for Neurodegeneration (CESNE), University of Padova, Padova, Italy; 11 Cogentech SRL Benefit Corporation, 20139 Milan, Italy; 12 IFOM-ETS AIRC Institute of Molecular Oncology, Milan, Italy; 13 Department of Cell Biology, Faculty of Medicine and Dentistry, University of Alberta, Edmonton, AB, Canada; 14 Padova Neuroscience Center, University of Padova, Padova, Italy; 15 APHP-Université Paris-Saclay, Reference Center for Neuromuscular Disorders Nord-Est-Ile de France, FILNEMUS, ERN-Euro-NMD, Creteil, France; 16 Pediatric Neurology and ICU Department, DMU Santé Enfant Adolescent (SEA), Raymond Poincaré University Hospital, Garches, France; 17 Venetian Institute of Molecular Medicine, Padova, Italy; 18 APHP, Reference Center for Neuromuscular Disorders Nord-Est-Ile de France, Neuromyology Department, Groupe Hospitalier Pitié-Salpêtrière, Paris, France

**Keywords:** ER stress, ERO1, SEPN1, TUDCA, multi mini-core disease, core myopathy

## Abstract

Selenoprotein N (SEPN1) is a protein of the endoplasmic reticulum (ER) whose inherited defects originate SEPN1-related myopathy (SEPN1-RM). Here, we identify an interaction between SEPN1 and the ER-stress-induced oxidoreductase ERO1A. SEPN1 and ERO1A, both enriched in mitochondria-associated membranes (MAMs), are involved in the redox regulation of proteins. ERO1A depletion in SEPN1 knockout cells restores ER redox, re-equilibrates short-range MAMs, and rescues mitochondrial bioenergetics. ERO1A knockout in a mouse background of SEPN1 loss blunts ER stress and improves multiple MAM functions, including Ca^2+^ levels and bioenergetics, thus reversing diaphragmatic weakness. The treatment of SEPN1 knockout mice with the ER stress inhibitor tauroursodeoxycholic acid (TUDCA) mirrors the results of ERO1A loss. Importantly, muscle biopsies from patients with SEPN1-RM exhibit ERO1A overexpression, and TUDCA-treated SEPN1-RM patient-derived primary myoblasts show improvement in bioenergetics. These findings point to ERO1A as a biomarker and a viable target for intervention and to TUDCA as a pharmacological treatment for SEPN1-RM.

## Highlights

• SEPN1 interacts with the endoplasmic-reticulum-stress-induced oxidoreductase ERO1A
• Upregulation of ERO1A is a hallmark of the SEPN1-RM preclinical models
• Targeting ERO1A improves the muscle disease phenotype of SEPN1-RM
• TUDCA, a pan ER stress inhibitor, is an effective pharmacological strategy for SEPN1-RM

## Graphical abstract

**Figure:**
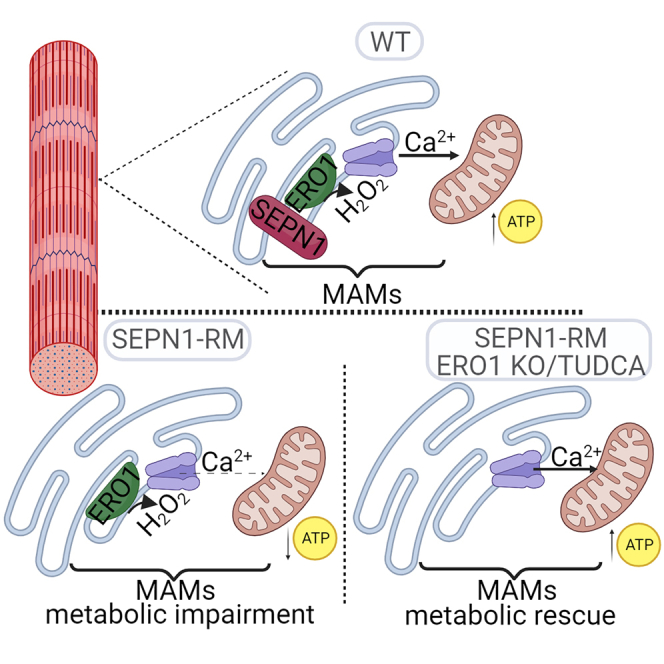

## Teaser

SEPN1-related myopathy (SEPN1-RM) is a congenital muscle disorder with no approved pharmacological treatment. Germani et al. show an interaction between SEPN1 and ERO1A and that targeting ERO1A improves the muscle phenotype of SEPN1-RM. Furthermore, they find that TUDCA, an FDA-approved drug, is an effective pharmacological strategy for SEPN1-RM.

## Introduction

Selenoprotein N (SEPN1 or SELENON) is a type II membrane protein of the endoplasmic reticulum (ER), acting as a calcium sensor in this organelle. It activates the calcium pump SERCA when ER calcium is low, through a redox activity.^1^^,^^2^^,^^3^ SEPN1 is enriched in a region of the ER in close contact with mitochondria, the so-called mitochondria-associated membranes (MAMs).^4^ This subdomain of the ER mediates ER-to-mitochondria calcium dynamics through the formation of calcium microdomains close to the mitochondria, thus allowing MAMs to control different cellular functions such as metabolism, organelle dynamics, and ER stress.^5^^,^^6^

Human recessive mutations in the *SEPN1* gene give rise to a congenital muscle disorder, generally referred to as SEPN1-related myopathy (SEPN1-RM), involving neck and trunk muscle weakness and atrophy, spinal rigidity, severe scoliosis, and diaphragmatic impairment leading to life-threatening respiratory failure.^7^^,^^8^ A particular characteristic of patients with SEPN1-RM is the severity of the paravertebral and diaphragmatic weakness compared to the relatively milder limb muscle involvement. Pediatric patients with SEPN1-RM develop severe scoliosis and respiratory failure due to diaphragmatic myopathy early in life; thus, they require systematic pulmonology studies and nocturnal non-invasive ventilation while still ambulant.^9^ The diaphragmatic weakness in patients with SEPN1-RM can be comparable to that in mitochondrial diaphragmatic myopathies. However, SEPN1 does not show any localization within mitochondria.^4^^,^^10^ Thus, at the moment, the pathogenesis and the molecular basis for the peculiar weakness of diaphragm and other muscles in SEPN1-RM remain elusive. As a result, there is no biomarker of the disease and no treatment beyond symptomatic management and life support.

Skeletal muscles generate reactive oxygen species during contraction and may undergo ER stress due to an excess of unfolded proteins.^11^ This stress triggers the unfolded protein response (UPR), which is mainly a homeostatic response that aims to re-establish a non-stress status in the organelle. The UPR is activated by three sensors, IRE1, PERK, and ATF6, localized at the ER membrane.^12^ The three sensors put corrective measures into action to re-establish proteostasis, consisting of a complex response of multi-layer signal transduction. This response activates the transcription of chaperones to enhance protein folding but, on the other hand, attenuates protein translation to eventually reduce the load of proteins to be folded. In skeletal muscle, the UPR is well characterized and helps to adapt this tissue to the increased energy requirements during exercise through metabolic rewiring.^13^ As an example, PERK, which is enriched at MAMs,^14^ boosts mitochondrial oxidative phosphorylation (OXPHOS) by promoting super-complex assembly.^15^

Among the mediators of the UPR, ERO1A (henceforth ERO1) is downstream to the PERK signal and is an intermediate catalyst of oxidative protein folding while also generating a stoichiometric amount of the oxidant H_2_O_2_.^16^^,^^17^ Also, ERO1 localizes at MAMs,^18^ and H_2_O_2_ nanodomains at the MAMs change mitochondrial activity, suggesting that ERO1-dependent H_2_O_2_ might impinge on bioenergetics as well.^19^ Of note, ERO1 modulates PERK, which boosts mitochondrial bioenergetics during ER stress.^20^

Previously, we have shown that SEPN1 loss impairs SERCA activity, which is further impaired by ERO1 activity, and that the deletion of ERO1’s transcription factor CHOP rescues the muscle weakness of SEPN1 knockout (KO) mice.^3^ However, the relationship between SEPN1 redox activity and the oxidative activity of ERO1, as well the consequence on the SEPN1-related muscle phenotype and the putative translational applications of this pathway, remains to be established.

Here, we show that ERO1 is consistently upregulated in preclinical models of SEPN1-RM. SEPN1 interacts covalently with ERO1, suggesting cross-regulation of their activities. SEPN1 loss impairs ER redox homeostasis and short-range MAMs involved in calcium dynamics between ER and mitochondria, leading to the consequent alterations in bioenergetics. In contrast, simultaneous SEPN1 and ERO1 loss restores redox balance, short-range MAMs, and bioenergetics. The absence of both proteins in a mouse context rescues the abnormalities in contacts between ER/sarcoplasmic reticulum (SR) and mitochondria, calcium dynamics, ATP, and diaphragm tension associated with the single SEPN1 loss. Along the same lines, *in vivo* treatment with the chemical chaperone tauroursodeoxycholic acid (TUDCA; by improving proteostasis) rescues calcium dynamics and diaphragm weakness in the SEPN1 KO mouse model and the ATP reduction in SEPN1-RM patient-derived primary myoblasts. These findings suggest that the couple SEPN1/ERO1 acts on MAM topology in a redox-tuned manner, regulating calcium dynamics, mitochondrial bioenergetics, and muscle function. Thus, these findings suggest ERO1 as a valuable biomarker of SEPN1-RM and the potential for targeted therapy with *ad hoc* chemical chaperone/ERO1 inhibitors for SEPN1-RM. Finally, this study shows an interaction between SEPN1 and ERO1, which might influence their respective activity; that ERO1 chronic loss in the diaphragm improves the muscle phenotype of the SEPN1-RM mouse model; and that we might repurpose TUDCA, an FDA-approved drug already in our pharmacological armamentarium, in SEPN1-RM preclinical models to speed clinical trial readiness.

## Results

### ERO1 as a biomarker of SEPN1-RM

**Figure 1:**
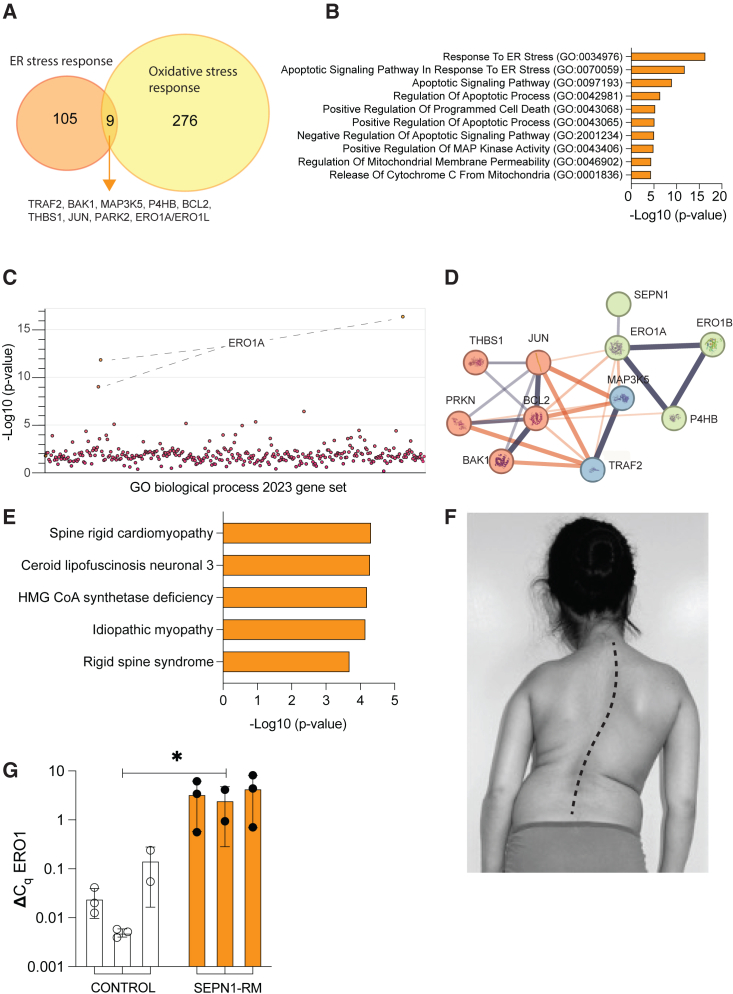
ERO1 a biomarker of SEPN1-RM (A) Venn diagram of intersecting target gene sets from ER and oxidative stress responses. (B) Top 10 processes, per the 2023 update, from GO analysis of common genes in (A). (C) Manhattan plot illustrating enrichment of input gene set in the 2023 GOBP gene set, with each point representing a single term. (D) Search tool for the retrieval of interacting genes/proteins (STRING) network analysis illustrates SEPN1’s connections with the nine specified genes (from A), revealing protein-protein functional and physical interactions based on published evidence compiled by STRING database (v.12). Each colored bubble signifies a functionally related gene cluster, with gray lines denoting intracluster connections and orange lines representing intercluster connections. The strength of the connections is indicated by the thickness of the connecting lines. (E) Top 5 diseases from the Rare Diseases AutoRIF database linked with the common gene subset from (A). (F) Phenotype of the SEPN1-RM patient P5 at age 15 years, showing amyotrophy of the upper limb proximal muscles (particularly deltoids) and the typical SEPN1-RM scoliosis, with dorsal and lumbar hyperlordosis and lateral trunk deviation. (G) Real-time RT-qPCR analysis of human ERO1 expression in cDNA samples derived from paravertebral muscle biopsies of three healthy controls and three patients with SEPN1-RM. Error bars denote SD; ∗p < 0.05 by Mann-Whitney test.

Functional studies on SEPN1 pointed to its role in modulating ER stress and oxidative stress response.^21^ Thus, in an attempt to find robust biomarkers of SEPN1-RM, we mined a public database provided by the Harmonizome to find overlapping target gene sets associated with ER stress (Gene Ontology [GO]: 0034976) (Table S1) and oxidative stress (GO: 0006979) (Table S1) response. We hypothesized that the gene products common to these two responses might be deregulated in the pathogenesis of SEPN1-RM and thus represent suitable biomarkers to facilitate therapeutic development for this disease (Figure 1A). Nine common genes (TRAF2, BAK1, MAP3K5, P4HB, BCL2, THBS1, JUN, PARK2, ERO1A/ERO1L) were enriched in the top two GO biological processes associated with ER stress response (Figure 1B). Moreover, we observed that among these nine genes, ERO1 is the common gene expressed in all top three significant GO biological processes associated with ER stress response (Figure 1C). In addition, we performed search tool for the retrieval of interacting genes/proteins analysis, based on the database compiling the protein-protein interactions from multiple published resources, indicating protein-protein interactions via physical interactions as well as functional associations. This analysis identified a link between SEPN1 and ERO1 within a local neighborhood cluster (Figure 1D). The observed network highlights that the queried protein’s interaction is in the primary layer with an average local clustering coefficient of 0.731 and an enrichment p value of 9.16e−09, suggesting that this set of protein interactions holds more significance than a randomly selected group of proteins with similar size and degree distribution from the genome. This enrichment suggests a biological connection among these proteins as a cohesive group. By applying the k-means clustering method to this network, the queried proteins are categorized into three clusters based on their closely related functions. Cluster 1 (Figure 1D, green bubble), linked with protein unfolding function, includes EROA1, ERO1B (or ERO1L), P4HB, and SEPN1, exhibiting an enrichment p value of 0.0091. Cluster 2 (Figure 1D, red bubble), associated with the UPR, involves BAK1, BCL2, JUN, PRKN, and THBS1, demonstrating an enrichment p value of 0.0306. Finally, cluster 3 (Figure 1D, blue bubble), connected with the IRE1-mediated UPR, encompasses MAP3K5 and TRAF2, with an enrichment p value of 0.0029. Interestingly, a subset of these genes, including ERO1, highlighted terms related to rigid spine and myopathy in the top Rare Diseases AutoRIF database through Enrichr R-package (Figure 1E). These findings suggest that ERO1 may serve as a robust biomarker linking SEPN1 mutations with ER stress and oxidative stress response.

To corroborate the data from the bioinformatics analysis and support ERO1 as a potential biomarker of SEPN1-RM, given that muscle dysfunction in this disease affects predominantly trunk muscles, we analyzed paraspinal muscle samples from three patients presenting with a classic, moderate-severity phenotype of SEPN1-RM (as indicated in Figure 1F and Table S2). All the patients had delayed motor development associated with muscle atrophy and weakness, which was moderate in proximal limb muscles and severe in neck and trunk muscles, leading to spinal rigidity (rigid spine syndrome), major scoliosis, and respiratory insufficiency from the end of the first decade of life; all required assisted ventilation while remaining ambulant (Figure 1F). Total RNA was extracted from snap-frozen paravertebral muscle biopsies and subjected to qPCR using primers targeting the human *ERO1*. A significant upregulation of *ERO1* expression was observed in all three patient samples when compared to controls (Figure 1G) for all the *SEPN1* mutations analyzed (missense variants in P3, frameshift variants in P4 and P5; Table S2) and independently of the type of histopathological lesion observed in the patient diagnostic biopsy. This supports the implication of *ERO1* in the pathophysiological mechanism of SEPN1-RM and confirms its relevance in the muscles most severely affected by the disease. It also suggests that *ERO1* might represent a target for therapeutic development in this disease and serve as a biomarker allowing us to measure target engagement and to monitor treatment response.

### SEPN1 and ERO1 functionally interact to modulate MAMs

**Figure 2:**
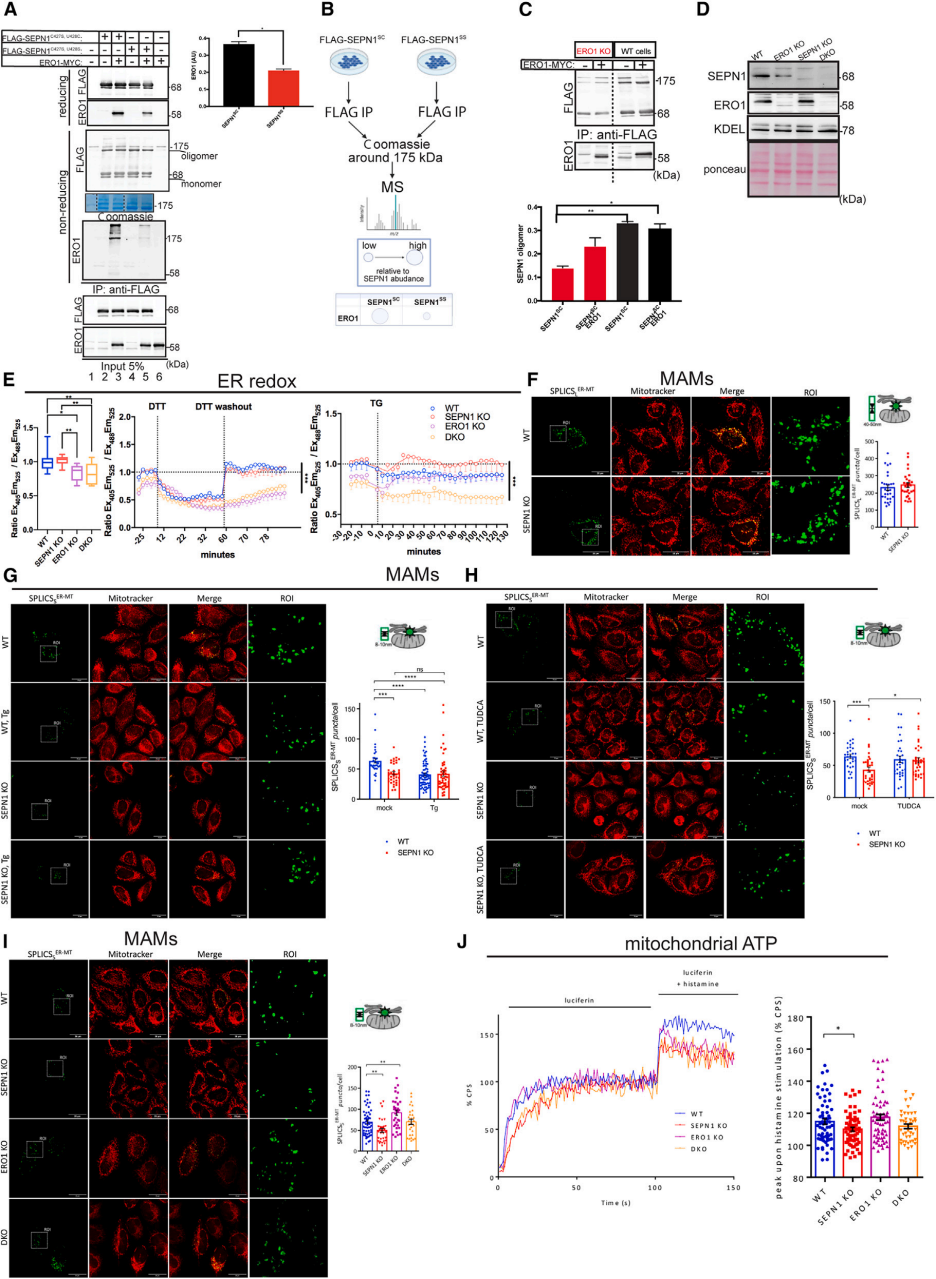
Interaction between SEPN1 and ERO1 impinges on short-distance MAMs and bioenergetics (A) FLAG and ERO1 immunoblots of FLAG-tagged SEPN1 immunopurified with FLAG-M2 antibody from lysate of cells that were untransfected or transfected with expression plasmids of the indicated proteins. The immunoprecipitates were resolved on reducing and non-reducing SDS-PAGE and Coomassie. On the right, a bar graph indicates the relative levels of ERO1 associated with its bait, FLAG-SEPN1 (n = 3, unpaired t test). The bottom images represent the 5% of the total input protein lysate immunopurified and resolved on reducing SDS-PAGE. (B) Scheme of the mass spectrometry analysis of FLAG-SEPN1 immunoprecipitates from HEK293T cells. The Coomassie band around 175 kDa was cut, digested, and analyzed by mass spectrometry. Spectra counts of ERO1 on those of SEPN1 in the same band are expressed as a ratio (low, high). (C) Detection of SEPN1 oligomers in WT and ERO1 KO HEK293T cells. FLAG immunoprecipitates were analyzed on non-reducing immunoblot. ERO1 immunoblot indicates the amount of ERO1 in the indicated samples. On the bottom is a bar graph indicating the ratio SEPN1 oligomers on total SEPN1 n = 3, one-way ANOVA). (D) ERO1 and SEPN1 immunoblot in WT, ERO1 KO, SEPN1 KO, and DKO HeLa cells. Immunoblot of KDEL containing proteins and Ponceau indicate protein loading. (E) Bar plots indicating the baseline fluorescence excitation ratio of roGFP2, reflecting the redox state of roGFP2 localized in the ER. On the right are traces of time-dependent changes in the fluorescence excitation ratio of roGFP2. Cells were exposed to a dithiothreitol (DTT; 1 mM) pulse of 20 min followed by a washout of the reductant and to 1 μM thapsigargin (Tg) treatment. Each data point represents the mean ± SEM of the fluorescence excitation ratio of roGFP2 (N = 8 fields of view per well obtained from two independent experiments, one-way ANOVA for repeated measures followed by Tukey’s multiple comparisons). (F) Images and quantification of SPLICS_L_^ER-MT^ in WT and SEPN1 KO HeLa cells (n = 3, unpaired t test). (G) Images and quantification of SPLICS_S_^ER-MT^ in WT and SEPN1 KO treated for 3 h with 1 μM Tg (n = 3, uncorrected Fisher’s least significant difference [LSD] test two-way ANOVA). (H) Images and quantification of SPLICS_S_^ER-MT^ in WT and SEPN1 KO treated for 24 h with 1 mM TUDCA (n = 3, uncorrected Fisher’s LSD test two-way ANOVA). (I) Images and quantification of short SPLICS in WT, SEPN1 KO, ERO1 KO, and DKO cells (scale bars: 25 μm). (J) Trace and quantification of ATP production in WT, SEPN1 KO, ERO1 KO, and DKO cells after stimulation with histamine. CPS indicates counts per second (Bonferroni multiple comparison after two-way ANOVA).

To investigate a physical interaction between SEPN1 and ERO1, and to identify whether SEPN1 was able to trap ERO1 in a redox-dependent manner, we used FLAG-tagged SEPN1^C427S, U428C^ , the redox-inert SEPN1^C427S, U428S^ (in which the redox-active U was replaced by a serine residue), and ERO1-MYC and expressed them in mammalian HEK 293T cells. Cells were harvested, and lysates were subjected to immunoprecipitation with FLAG-M2 antibody. Immunoblot analysis in non-reducing conditions showed, as expected, that SEPN1 self-associates, forming oligomeric species around 175 kDa (lanes 2–5), and that FLAG-SEPN1^C427S, U428C^ associates covalently with ERO1 in a complex, which migrates at a molecular size close to that of the SEPN1 oligomer (lane 3) (Figure 2A). Mass spectrometry analysis of the band running at 175 kDa confirmed the prevalent association between FLAG-SEPN1^C427S, U428C^ and ERO1 in a high-molecular-weight complex with a number of spectra counts that was double those found in the FLAG-SEPN1^C427S, U428S^ immunoprecipitation, in accordance with the immunoblot signal, and indicating that the covalent interaction between SEPN1 and ERO1 is mediated by C428 in SEPN1 (Figure 2B).

Next, we investigated the biological significance of the SEPN1-ERO1 interaction. The SEPN1 disulfide-bonded oligomeric state was detected through immunoprecipitation and non-reducing immunoblot in cells with or without ERO1. This assay indicated that the SEPN1 oligomeric state is mediated by ERO1 (which might be engaged in an oligomer with SEPN1 and other proteins in its turn), and in fact, the SEPN1 oligomeric state was strongly impaired in cells lacking ERO1 (Figure 2C). Therefore, the interaction between SEPN1 and ERO1 promotes SEPN1 oligomerization, which, as shown by our earlier experiments, impairs SEPN1’s redox-trapping potential.^1^ To track ER redox poise and identify differences among wild-type (WT), SEPN1 KO, ERO1 KO, and double (SEPN1, ERO1) KO (DKO) HeLa cells (Figure 2D), we used the ratiometric redox sensor ER-localized roGFP2.^22^^,^^23^ The redox changes of the sensor in live cells were measured by comparing sensor emission intensity at 525 nm when excited at 405 (excitation [Ex]_405_emission[Em]_525_) and 488 (Ex_488_Em_525_) nm. The oxidation within ER was slightly decreased in ERO1 KO, as was also suggested previously,^24^ and in DKO cells. ER roGFP2 was reduced after a challenge with the reductant dithiothreitol in the cells with the four different genotypes; however, during the washout from the reductant, both ERO1 KO and DKO cells were slower to recover the baseline oxidative poise, suggesting that, as expected, the cells without ERO1 stayed reduced longer, and the simultaneous lack of SEPN1 did not produce any further effect on the reduced ER redox poise (Figure 2E). As previously reported,^1^ thapsigargin (Tg), an inhibitor of SERCA, caused a progressive reduction of the redox sensor in WT cells, while SEPN1 KO cells were not responsive, but DKO cells acquired back the reductive shift upon Tg such as WT cells. This suggests that the contemporary lack of SEPN1 and ERO1 increased the reduced poise of the ER lumen after calcium depletion, similar to what happens in WT cells, thus indicating an advantage of the lack of the two proteins in adjusting the reduced lumen’s redox poise to calcium levels (Figure 2E).

As both SEPN1 and ERO1 are enriched in MAMs,^4^^,^^20^^,^^25^ we investigated whether both proteins affected MAMs using split GFP-based contact site sensors (long and short SPLICS).^26^ There was no difference between WT and SEPN1 KO cells in long-distance MAMs (40–50 nm) (Figure 2F), but there was a decrease in short-distance MAMs (8–10 nm) in SEPN1 KO cells (Figure 2G). Interestingly, a short treatment with Tg, which blunts calcium uptake in the ER, reduced the short-distance MAMs of WT cells, but there was no further decrease in SEPN1 KO cells, indicating that calcium levels in the ER might affect these short-distance contacts, and this phenotype was paralleled by SEPN1 loss (Figure 2G). The decrease in short-distance MAMs was confirmed in SEPN1 knockdown (KD) of the myoblast cell line C2C12^2^ (Figure S1A, related to Figure 2). SEPN1 KD myoblasts cultured in a medium with low glucose displayed an increase of ERO1 and of the ER stress marker BIP, suggesting an ongoing ER stress (Figures S1B and S1C, related to Figure 2). Treatments of SEPN1 KD with EN460, a known ERO1 inhibitor,^27^ or with the chemical chaperone (pan-ER stress inhibitor) TUDCA improved SEPN1 KD cell viability (Figure S1D, related to Figure 2). Given the potential toxicity of EN460, we proceeded with TUDCA alone. TUDCA rescued also the defect in short-distance MAMs in SEPN1 KO cells, indicating the beneficial effect of the restored proteostasis on these contacts (Figure 2H). In quantitative agreement with TUDCA-treated SEPN1 KO cells, the DKO cells had an increased number of the short-distance MAMs, suggesting the beneficial effect of the simultaneous loss of SEPN1 and ERO1 on the recovery of these contacts (Figure 2I).

As short-distance MAMs are involved in bioenergetics,^28^^,^^29^ we measured mitochondrial ATP production in a time-lapse setting in WT, SEPN1 KO, ERO1 KO, and DKO cells after IP3R stimulation through its agonist histamine. ATP levels were lower in SEPN1 KO cells than in WT cells, but the levels in ERO1 KO or DKO cells were no different from WT (Figure 2J).

Overall, these findings indicate that from a bioenergetic point of view, SEPN1 loss is deleterious, but the double loss of SEPN1 and ERO1 corrects the defect.

### ERO1 genetic inhibition in SEPN1 KO diaphragm rescues UPR and bioenergetics

**Figure 3:**
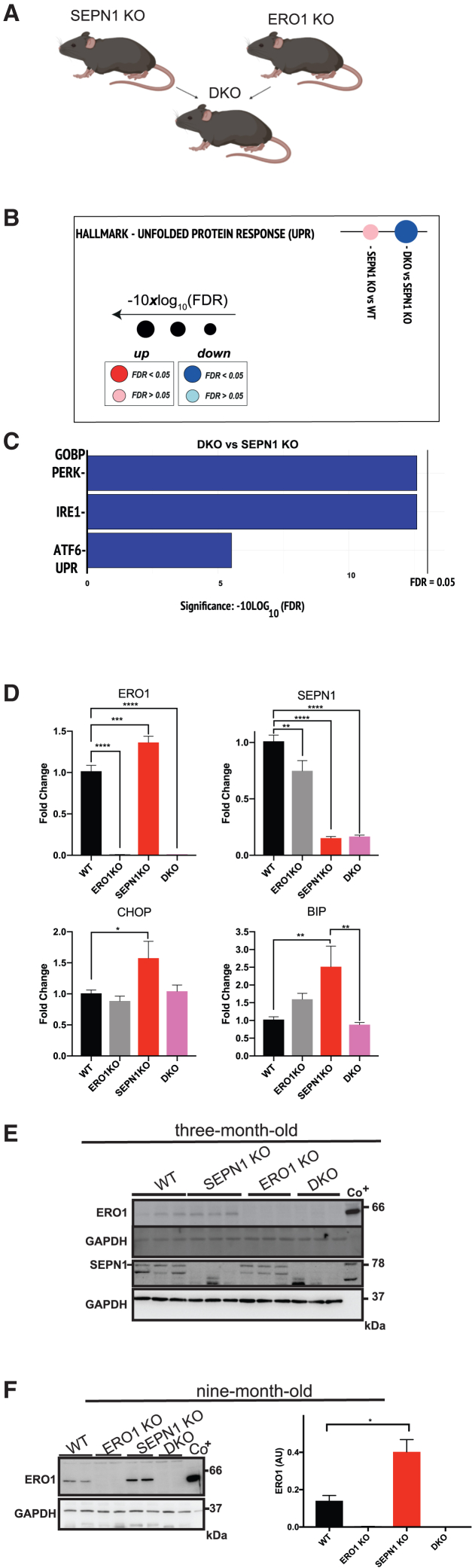
UPR induction in SEPN1 KO diaphragms is rescued in DKO counterparts (A) Graphical representation of the cross between SEPN1 KO and ERO1 KO mice to get (SEPN1, ERO1 KO) DKO mice. (B) Dot plots in hallmark gene sets indicating the upregulation of UPR in SEPN1 KO diaphragms (pink/red dots) and the downregulation in DKO (light blue/blue dots) from 9-month-old mice (n = 4). (C) Bar plots indicating downregulation (blue bars) of PERK-, IRE1-, and ATF6-mediated UPR pathways by GOBP in the DKO when compared to SEPN1 KO. FDR indicates false discovery rate. (D) Real-time qPCR on cDNA from diaphragms of mice of the genotypes indicated (n = 5, one-way ANOVA). (E) ERO1 and SEPN1 immunoblot from 3-month-old mice. GAPDH immunoblot indicates equal protein loading. (F) ERO1 immunoblot from 9-month-old mice. GAPDH immunoblot indicates equal protein loading. On the right is a bar graph indicating ERO1 levels in arbitrary units (a.u.) (n = 4, unpaired t test).

To clarify the molecular processes underlying the alterations in SEPN1 KO diaphragms and the difference in these processes due to lack of ERO1, we developed double SEPN1 and ERO1 KO (DKO) mice after crossing SEPN1 KO mice with ERO1 KO mice (Figure 3A).^30^ RNA sequencing analysis was done on diaphragms of young (2–9 months old) and old (18 months old) SEPN1 KO mice, together with age-matched WT and ERO1 KO as well as DKO siblings. As previously, we identified a functional compensation in the diaphragms of double SEPN1, CHOP, KO (DKO_2_)^3^; we also did RNA sequencing analysis on RNAs from these diaphragms (together with age-matched CHOP KO). Gene set enrichment analysis (GSEA) identified the UPR as tendentially upregulated in SEPN1 KO diaphragms and significantly downregulated in DKO (false discovery rate < 0.05) (Figures 3B, S2A, and S2B). The evaluation of PERK-, IRE1-, and ATF6-mediated UPR pathways by GO Biological Process indicated a downregulation of all three UPR branches in DKO when compared to SEPN1 KO (Figure 3C). Real-time qPCR on diaphragm transcripts from 9-month-old SEPN1 KO mice confirmed the upregulation of UPR markers ERO1, CHOP, and BIP, while levels of CHOP and BIP were quantitatively similar between WT and DKO diaphragms (Figure 3D).

**Figure 4:**
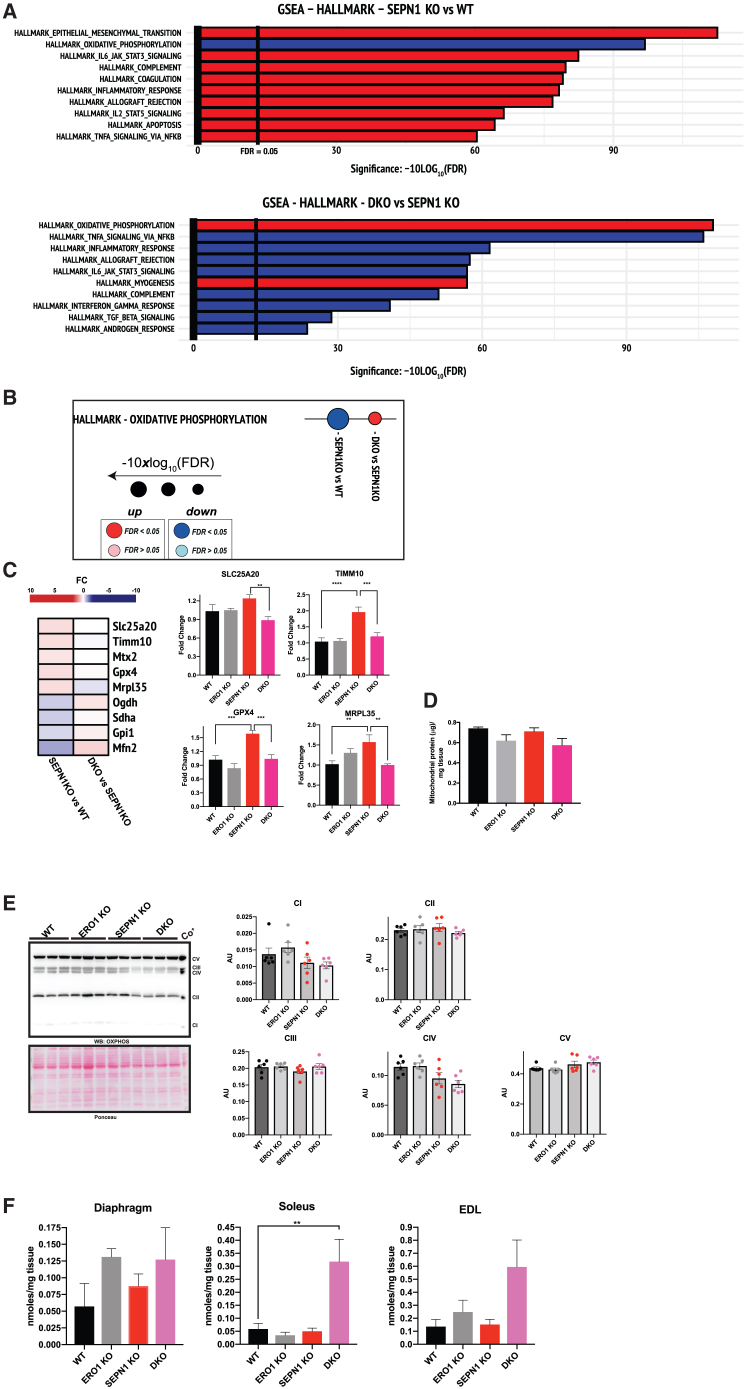
Altered OXPHOS in diaphragms of SEPN1 KO mice is regularized in DKO mice (A) Bar graphs indicating the top ten most perturbed gene sets (hallmark) of SEPN1 KO and DKO diaphragms. Enrichment and their FDR-adjusted p values were computed using a camera (preranked) and were determined on the hallmark gene sets collection. The x axis reports the logarithmically transformed FDR in the form of −10×log10 (FDR), with a bold intercept (x = 13.01) indicating the FDR threshold of 0.05. Red bars: upregulated; blue bars: downregulated. (B) Dot plots in hallmark gene sets indicating the down- (blue dots) and upregulation (red dots) of OXPHOS in SEPN1 KO and DKO diaphragms, respectively (n = 4). (C) Heatmap of OXPHOS genes from the hallmark gene sets collection differently regulated in SEPN1 KO and DKO diaphragms. On the right are bar graphs indicating results from real-time qPCR on cDNA from diaphragms of mice of the genotypes indicated (n = 5, one-way ANOVA). (D) Bar graphs indicating mitochondrial protein content of the diaphragms after isolation of pure mitochondria (Mito;n = 3, one-way ANOVA). (E) Immunoblot of the different complexes (I–V) of OXPHOS in diaphragms of the indicated genotypes. On the right are quantifications of the complexes. (F) ATP levels in diaphragm, soleus, and EDL muscles (n = 5, one-way ANOVA).

Immunoblot analysis of proteins from diaphragms of 3-month old mice failed to detect any induction of ERO1 expression in SEPN1 KO mice (Figure 3E), but ERO1 protein amounts were double in diaphragms from 9-month-old SEPN1 KO mice, suggesting an induction of ERO1 expression in SEPN1 KO diaphragms over time (Figure 3F). Interestingly, GSEA identified OXPHOS as the pathway most downregulated in SEPN1 KO diaphragms and was instead upregulated in the age-matched DKO counterparts (Figures 4A and 4B). This finding was corroborated by the fact that in DKO_2_ diaphragms, OXHOS was upregulated too (Figure S2C). Transcripts belonging to OXPHOS and expressed differently in SEPN1 KO and DKO diaphragms (e.g., SLC25A20, TIMM10, GPX4, and MRPL35) were analyzed also by real-time qPCR and were altered in SEPN1 KO but were quantitatively similar in WT, ERO1 KO, or DKO (Figure 4C). These observations indicated UPR upregulation in SEPN1 KO diaphragms but its downregulation in DKO and that, conversely, the expression of the OXPHOS pathway is downregulated in SEPN1 KO diaphragms but upregulated in DKO. Although the mitochondrial protein content (Figure 4D) and the proteins of the complexes of the respiratory chain were quantitatively similar in the diaphragms of the different genotypes (Figure 4E), ATP increased in all muscles (diaphragm, soleus, and extensor digitorum longus [EDL]; although the difference was statistically significant only in soleus) of DKO mice (Figure 4F), suggesting potentiated bioenergetics in DKO muscles.

### Structural abnormalities in the Ca^2+^ release unit (CRU)/mitochondria couple and impaired function of the SEPN1 KO diaphragm is rescued by ERO1 genetic inhibition

**Figure 5:**
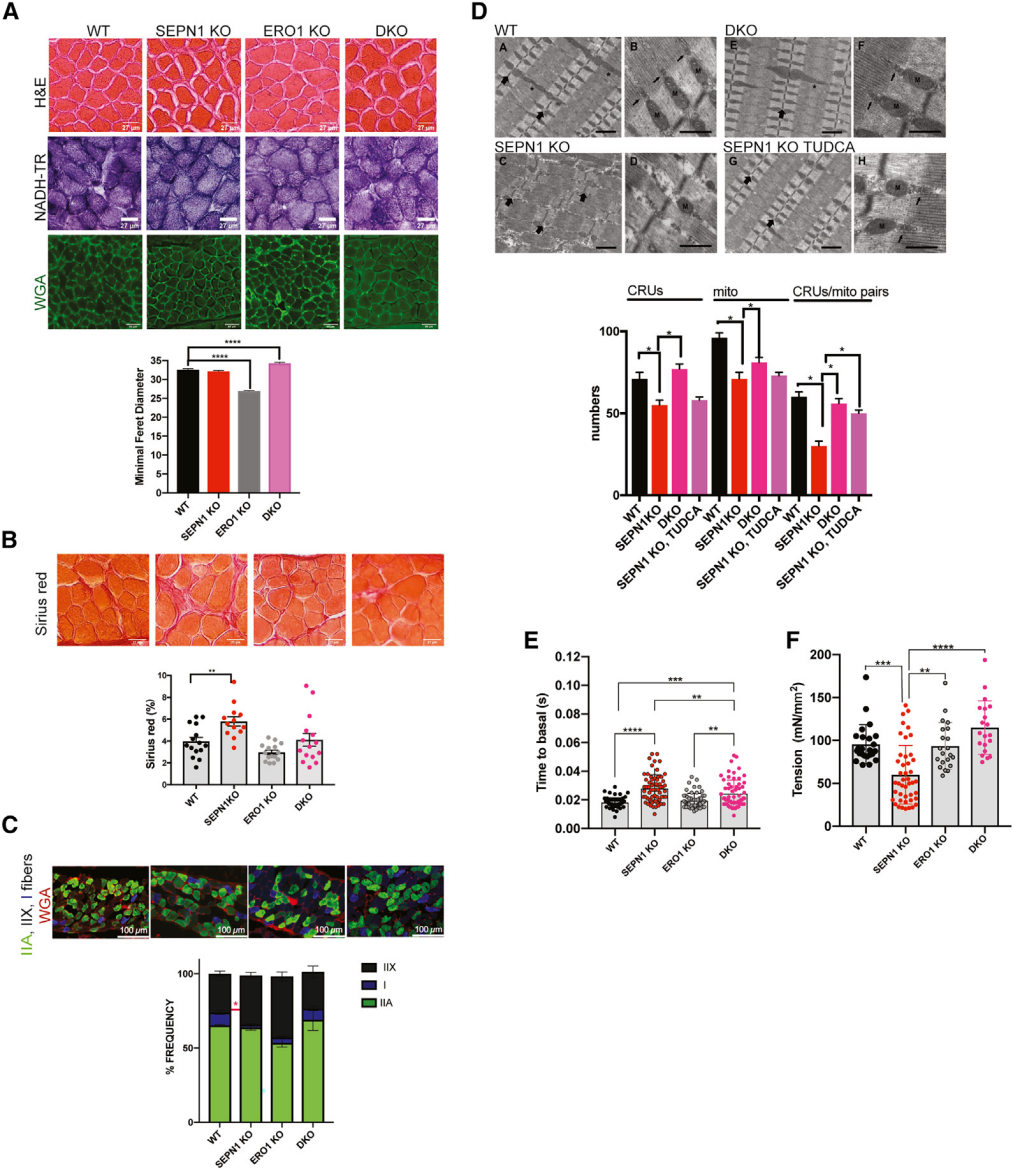
Defects in CRU-to-Mito apposition of SEPN1 KO mice are rescued by ERO1 loss and TUDCA (A) Hematoxylin and eosin (H&E), nicotinamide adenine dinucleotide tetrazolium reductase (NADH-TR), and wheat germ agglutinin (WGA) staining of representative transverse frozen sections of diaphragms from 6-month-old mice. On the bottom is a histogram of the relative frequency of the minimal Feret diameter of the diaphragm fibers. (B) Sirius red staining of transverse frozen sections of diaphragms. On the bottom are dot plots indicating Sirius red-positive areas as percentages. (C) Representative images of fast (IIA and IIX)- and slow (I)-twitch muscle fibers in diaphragm muscle sections using immunostaining with anti-fast and anti-slow MyHC antibodies. Below, the bar graph shows the percentages of fiber types (n = 5 mice/genotype, one-way ANOVA). (D) Representative EM images from WT (A and B), SEPN1 KO (C and D), DKO (E and F), and SEPN1 KO, TUDCA (G and H) diaphragms, respectively. Labeling: large arrows point to the Z line; small arrows point to CRUs or triads; asterisk is for longitudinal A-band; Mito and M is for mitochondria. Scale bars: (A, E, C, and G) 1 μm and (B, F, D, and H) 0.5 μm. On the bottom is quantification of CRU, mito (mitochondria), and CRU/mito pairs. Data are expressed as average number per 100 μm^2^ and shown as mean ± SEM (n = 3, One-way ANOVA followed by post-hoc Tukey test for multiple comparisons) (E) Time to 50% basal measurement of calcium uptake after single-pulse stimulation in primary culture of mouse FDB fibers. (F) Effect of recovery of diaphragm single-fiber tension (n = 5 mice/genotype, non-parametric one-way ANOVA, Kruskall-Wallis multiple comparison test).

Hematoxylin and eosin and nicotinamide adenine dinucleotide tetrazolium reductase staining, together with quantification of mitochondria protein contents of diaphragms of the four aforementioned genotypes, ruled out gross muscle fiber alterations, defects in the oxidative enzyme, or differences among the different genotypes (Figures 4D and 5A). However, quantification of fibers by wheat germ agglutinin (WGA) staining showed a higher relative frequency in minimal Feret diameter of DKO diaphragms, suggesting bigger fibers (Figure 5A). The quantification of Sirius red staining in Figure 5B indicated that SEPN1 KO diaphragms showed a steep increase in fibrotic tissue that was less important in DKO. We performed immunohistochemistry with fiber-type-specific myosin heavy chain (MyHC) antibodies (IIA, IIX, and I) to examine the diaphragm’s fiber-type proportion. We noticed a slight decrease in slow (oxidative) type I fibers in SEPN1 KO that, however, given the low level of type I fibers (<8%) and the potential presence of hybrid fibers, ruled out any overt fiber-type switch (Figure 5C).

In electron microscopy (EM) longitudinal sections of diaphragm fibers from WT mice, mitochondria usually appear as small round/oval profiles and are mainly located within the sarcomere I band symmetrically positioned on either side of the Z line (Figure 5DA, large arrows) closely opposed to the junctional SR of the CRU or triads (Figure 5DB, small arrows); these are referred to as triadic mitochondria. In type I and type IIA fibers, in addition to the I-band triadic mitochondria, a smaller subset of mitochondria with a more variable size/shape are longitudinally positioned between myofibrils and span the entire sarcomere A-band length (Figure 5DA, asterisks). However, in diaphragms from SEPN1 KO of young and old mice, we noted localized areas with few or almost absent mitochondria within the intermyofibrillar spaces in about 30% of the fibers (Figures 5DC and 5DD, large arrows). Interestingly, higher magnification allowed us to detect that in the free mitochondria areas, the CRUs were also usually missing (Figure 5DD). Abnormal accumulations of mitochondria under the sarcolemma (Figure S2F, empty arrow) or into quite long intermyofibrillar rows (Figures S2DE–S2DG) were often present in diaphragms from old SEPN1 KO mice, suggesting a translocation and/or redistribution of mitochondria from their correct triadic position. In contrast, analysis and quantification of ultrastructural changes in diaphragm fibers from DKO and DKO_2_ mice revealed an ultrastructural disposition/organization of mitochondria and CRUs apparently similar to that of WT diaphragms (Figures 5DE, 5DF, and S2 A–S2D; Table S3).

The quantitative ultrastructural analysis supported the correct topology of mitochondria and their association with the CRU in DKO diaphragms, reflecting a rescued muscle phenotype.

From a functional point of view, the relaxation time (time to basal 50%) of SEPN1 KO flexor digitorum brevis (FDB) fibers after a pulse stimulation was significantly increased but partially lowered in DKO and DKO_2_ (Figures 5E and S2E), suggesting a partial recovery in calcium dynamics of DKO and DKO_2_.^3^ Interestingly, the tension developed by skinned fibers of SEPN1 KO mice diaphragms was significantly lower than that of WT, suggesting alterations in the core contractile apparatus of single muscle fibers lacking SEPN1. However, this fiber dysfunction was completely prevented by the elimination of ERO1 or CHOP in SEPN1 KO mice (respectively, DKO and DKO_2_) (Figures 5F and S2F).

Thus, these findings are consistent with a functional improvement of muscle performance in SEPN1 KO mice as a result of ERO1 genetic inhibition.

### TUDCA rescues the calcium dynamics and diaphragmatic weakness of SEPN1 KO mice

**Figure 6:**
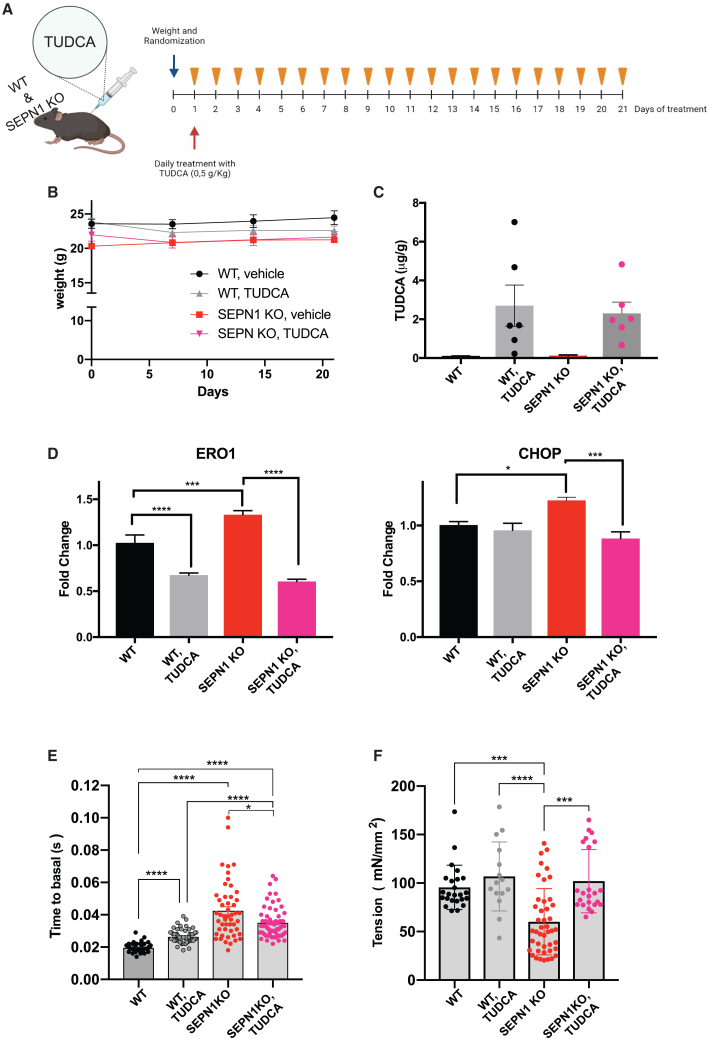
TUDCA-mediated improvement of SEPN1 KO muscle phenotype (A) Treatment scheme of TUDCA in WT and SEPN1 KO mice. (B) Weekly weights of mice during treatment. (C) Dot plots indicating the TUDCA levels in diaphragms of WT and SEPN1 KO mice after TUDCA treatment or placebo (n = 6). (D) Real-time qRT-PCR analysis of ERO1 and CHOP from mRNA from diaphragms (n = 5, one-way ANOVA). (E) Time to 50% basal measurement of calcium uptake after single-pulse stimulation in primary culture of mouse FDB fibers. (F) Effect of recovery of diaphragm single-fiber tension (n = 10 mice/genotype, one-way ANOVA, Kruskall-Wallis multiple comparison test).

To address whether the SEPN1 KO muscle phenotype might be rescued by reducing ERO1-mediated proteotoxicity and ER stress through a pharmacological intervention, we tested the chemical chaperone TUDCA. Five-month-old WT and SEPN1 KO mice were intraperitoneally administered with 0.5 g/kg TUDCA daily over a period of 21 days and compared to placebo-treated mice (Figure 6A). During TUDCA treatment, mice stayed healthy with a good appearance and did not show any overt weight loss, thus ruling out any side effects of the treatment (Figure 6B). Twenty-four hours after the last dose, plasmatic TUDCA levels were 0.19 ± 0.10 and 0.29 ± 0.15 μg/mL in WT and SEPN1 KO mice, respectively, not significantly different from the basal TUDCA values measured in placebo-treated mice (0.30 ± 0.10 μg/mL). These data confirmed our preliminary observation, suggesting no accumulation in the plasma with daily TUDCA administration. On the contrary, accumulation occurs in diaphragms, as indicated by the significantly higher TUDCA levels in this muscle in comparison to those in placebo-treated mice (Figure 6C). Consistent with its presence in diaphragm, TUDCA treatment alleviated ER stress in this muscle, as indicated by reductions in ERO1 and CHOP levels in the diaphragms of WT and SEPN1 KO mice (Figure 6D). Importantly, this treatment improved calcium dynamics in FDB muscle fibers (Figure 6E) and diaphragm single-fiber tension (Figure 6F) compared to placebo-treated mutant mice. Thus, treatment of TUDCA for 3 weeks was sufficient to restore force production in single fibers from SEPN1 KO mice, showing that SEPN1-related dysfunction of the contractile apparatus can be completely reversed.

To understand whether the improved tension in treated SEPN1 KO mice correlated with the rescue of the CRU/mitochondria couples, we used EM to quantify the number of these couples on ultrathin diaphragm sections. The rate of couples was recovered with TUDCA as happened in DKO and DKO_2_ diaphragms (Figures 5DG and 5DH and in Table S3). Thus, these findings are consistent with a TUDCA-mediated functional improvement of muscle performance in SEPN1 KO mice.

### TUDCA rescues the bioenergetics defect in myoblasts from patients with SEPN1-RM

**Figure 7:**
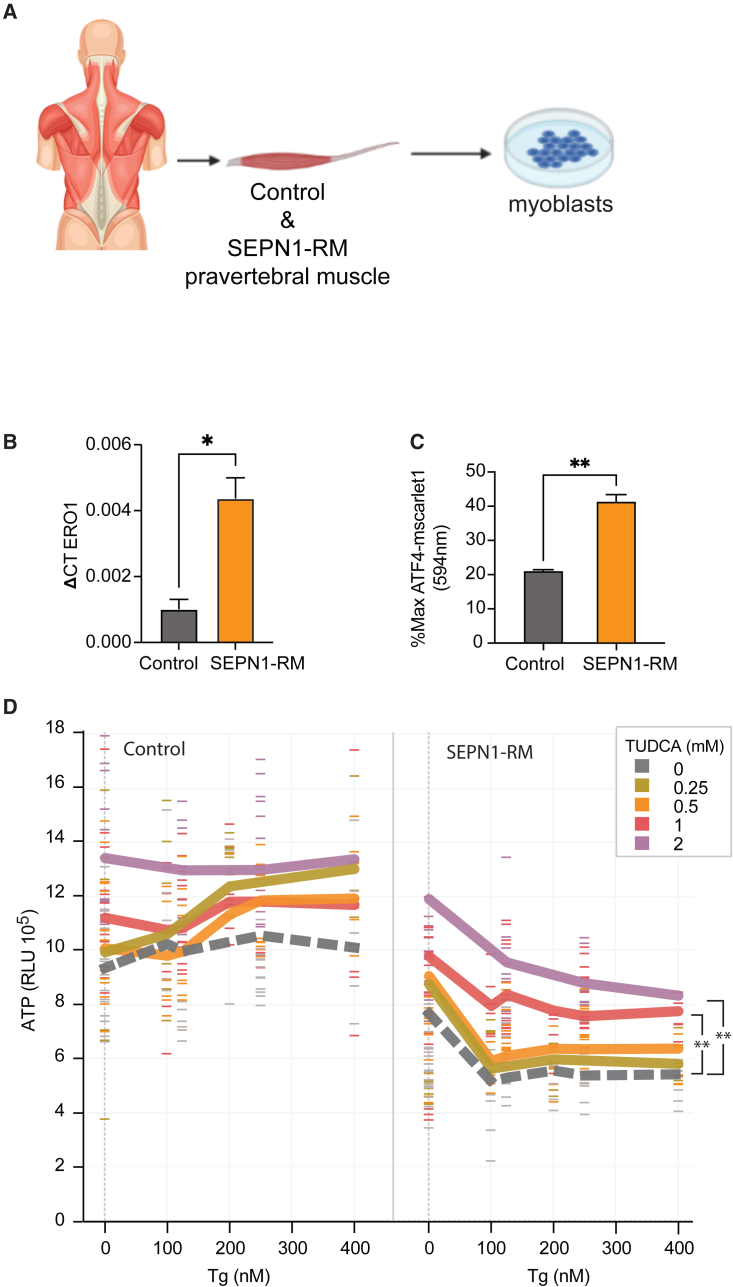
ER stress associated with ATP deficit in human SEPN1-RM primary myoblasts rescued by TUDCA treatment (A) Graphical representation of myoblast isolation in patients with SEPN1-RM. (B) Real-time RT-qPCR analysis of ERO1 expression in SEPN1-RM myoblasts relative to age- and passage-paired healthy myoblast controls. (C) Level of ATF4-mScarlet fluorescent signal with respect to each maximum (594 nm) 24 h post-H_2_O_2_ treatment (positive signal control). (D) ATP level (relative luminescence unit, RLU) in human myoblast cultures treated with increasing doses of Tg (0–400 nM) and TUDCA (0, 0.25, 0.5, 1, and 2 mM). Line plot shows the running average with the overlaid scatterplot indicating the range of ATP levels observed in each condition (n = 5, two-way ANOVA with Dunnett post hoc correction).

Next, to examine whether ER stress/UPR, accompanied by an upregulation of ERO1, was a common finding of SEPN1-RM preclinical models, we examined primary myoblasts from three patients with SEPN1-RM with a classic, moderate phenotype as described above and expressing different nonsense and missense *SEPN1* mutations (Table S2; Figure 7A). ERO1 expression was upregulated roughly four times compared to healthy controls in SEPN1-RM myoblasts (Figure 7B). The levels of ATF4, another mediator of the UPR, were also twice as high compared to controls (Figure 7C). These findings suggest an ongoing UPR with attendant high levels of ERO1 in SEPN1-RM patient myoblasts. The UPR and ERO1 upregulation were associated with reduced ATP levels in SEPN1-RM myoblasts (Figure 7D). Importantly, TUDCA improved the ATP levels of this set of SEPN1-RM myoblasts both under basal conditions and after exposure to increasing concentrations of Tg, suggesting that TUDCA efficiently enhances the bioenergetics of SEPN1-RM (Figure 7D). Consequently, TUDCA could be an effective therapeutic option for patients with SEPN1-RM.

## Discussion

Therapeutic intervention for SEPN1-RM is currently limited to palliative measures; incomplete understanding of its molecular mechanism and lack of biomarkers hinder therapeutic development in this rare genetic disease.^21^ Here, we identify a physical interaction between SEPN1 and the ER-stress-regulated oxidoreductase ERO1, mediated by a covalent binding. ERO1 promotes the formation of SEPN1 oligomers bound together via disulfide bonds—a state that may impact SEPN1 functionality. The self-association between SEPN1 protomers is in fact mediated by non-covalent interactions, and also by a disulfide bridge involving C108, suggesting a role of ERO1 in the redox state of SEPN1 and thus its activity.^1^^,^^16^ Functionally, both ERO1 and SEPN1 play critical roles in maintaining the ER redox balance. In their absence, this equilibrium is disrupted, resulting in a more “reduced” state within the ER. This suggests a broad redox role for the combined action of SEPN1 and ERO1 on ER proteins, probably not only restricted to the calcium handling proteins SERCA, RYR, and IP3R^1^^,^^31^^,^^32^^,^^33^ but also involving tethers between ER and mitochondria.^34^

Both ERO1 and SEPN1 are enriched in MAMs, a contact region between ER and mitochondria,^4^^,^^25^ and their loss impacts on these contacts. Here, we show that SEPN1 loss selectively impairs short-range MAMs, while ERO1 loss tightens them. Despite the ubiquitous expression of these two proteins, loss of function of SEPN1 in humans gives rise to a myopathic phenotype involving mainly oxidative muscles such as the diaphragm and paravertebral muscles.^35^ Previously, we identified fewer contacts between the CRUs and mitochondria, together with mitochondria delocalization at the A band on muscle biopsies from patients with SEPN1-RM.^4^ Here, despite the apparently mild muscle phenotype of SEPN1 KO mice, we counted fewer contacts between CRUs and mitochondria in their diaphragms, suggesting that blunted CRU/mitochondria contacts are a characteristic feature of SEPN1-deficient models. Interestingly, the simultaneous loss of ERO1 and SEPN1 decreases ER oxidative poise while rescuing the reduction in the aforementioned contacts in both cell lines and diaphragms. It is still a matter of speculation whether the interaction between SEPN1 and ERO1 acts directly on the MAMs by redox-regulating proteinaceous tethers including SEPN1 itself or the effects on MAMs are secondary to those on calcium handling through the formation of calcium microdomains between ER and mitochondria. This latter hypothesis raises the possibility that calcium microdomains per se might regulate the distance between ER and mitochondria.^29^ The effect of SEPN1/ERO1 interaction on calcium microdomains in MAMs might control mitochondrial metabolism through OXPHOS by activating enzymes of the Krebs cycle and ATP production by stimulating ADP transporter and complex V.^36^ However, H_2_O_2_ nanodomains in MAMs modulate OXPHOS in mitochondria,^19^ and hyper-oxidation of components of the excitation-contraction coupling machinery impairs muscle force generation.^37^ Thus, it is conceivable that, besides Ca^2+^, H_2_O_2_ from ERO1 oxidative activity in MAMs can modulate mitochondria bioenergetics and muscle force as well, and indeed, SEPN1-RM was associated with hyper-oxidation and oxidative stress.^35^^,^^38^^,^^39^ Despite the unknown culprit (Ca^2+^ or H_2_O_2_), RNA sequencing analysis on diaphragms unequivocally suggests that the expression of genes encoding the OXPHOS process is dysregulated in young and old SEPN1 KO mice. Hence, the chain of events connects an impairment in redox homeostasis and MAMs with the downregulation in OXPHOS, culminating in reduced diaphragmatic tension in SEPN1 KO mice.

Furthermore, we detected an UPR with an attendant increase in ERO1 levels in SEPN1 KO diaphragms, consistent with ERO1 overexpression in SEPN1-RM patient muscles, and suggesting global ER stress/UPR in the absence of SEPN1. UPR mediators, such as PERK, act on MAMs, regulating ER to mitochondrial calcium flux and OXPHOS.^15^^,^^40^^,^^41^ Thus, components of the UPR other than ERO1 in SEPN1 KO diaphragms might trigger the cascade of events connecting MAM function with mitochondrial bioenergetics. However, the simultaneous loss of SEPN1 and ERO1 (or the upstream CHOP) downregulates the UPR and tightens MAMs, improving ER-mitochondrial calcium transfer and increasing OXPHOS, thus highlighting a central role of ERO1 in the UPR and MAMs in SEPN1 KO diaphragms. SEPN1-RM therefore arises from a defect in redox proteostasis accompanied by an ERO1 surge, leading to chronic ER stress**,** UPR, functional impairment of MAMs, and, consequently, compromised mitochondrial bioenergetics and diaphragmatic tension.

These findings have direct translational implications for therapeutic development in SEPN1-RM. Given the lack of ERO1 inhibitors without off-target effects^27^ and on the basis of persistent ER stress/UPR in diaphragms of SEPN1 KO mice, we tested the chemical chaperone/ER stress inhibitor TUDCA for its ability to recover proteostasis and, hence, to improve SEPN1-RM muscle function. TUDCA is a natural product, a bile acid produced in the liver, and is approved by the FDA for the treatment of chronic cholestatic liver diseases and for gallstones.^42^^,^^43^ Clinical studies on patients report that chronic treatment with hydrophilic bile acids is well tolerated, with no substantial side effects, suggesting a good safety profile.^44^ From a functional point of view, TUDCA reduces ER and oxidative stress, acting as a chemical chaperone to protect mitochondria, and has anti-apoptotic and cytoprotective effects in animal models of neurodegenerative and non-neurodegenerative disease.^44^^,^^45^ Ongoing clinical trials are assessing TUDCA’s potential in treating amyotrophic lateral sclerosis, Parkinson disease, Alzheimer disease, and multiple sclerosis.^44^^,^^46^ Furthermore, TUDCA reduces insulin resistance in humans, which is a feature of some patients with SEPN1-RM.^47^^,^^48^ *In vivo* administration of TUDCA in SEPN1 KO mice for 3 weeks rescued the calcium handling defect of muscle fibers and that of the impaired diaphragmatic tension, indicating overall improvement in excitation-contraction coupling. Experiments on TUDCA-treated myoblasts from three patients with SEPN1-RM expressing different SEPN1 mutations also indicate an improvement in ATP levels in basal conditions and under ER stress, indicating a rescue of the bioenergetics deficiency affecting SEPN1-RM myoblasts. While these results need to be confirmed with a larger number of *SEPN1* mutations, TUDCA-mediated rescue of the bioenergetic defect in myoblasts from patients with SEPN1-RM confirms its clinical importance and suggests the feasibility of repurposing TUDCA for patients with SEPN1-RM. Finally, our study emphasizes the role of ERO1 in the pathogenesis of SEPN1-RM. Upregulation of ERO1 in both frozen muscle samples and primary cultured myoblasts from patients with SEPN1-RM with different mutations confirms ERO1 implication in the disease. Further studies are needed on SEPN1-RM biopsies and will clarify a putative correlation between ERO1 overexpression and disease severity. However, our findings suggest that ERO1 might be useful as a biomarker, allowing us to measure target engagement and monitor treatment response and thereby paving the way for therapeutic development in SEPN1-RM. Additionally, ERO1 represents a relevant therapeutic target; indeed, our data support the interest in the clinical development of a selective ERO1 inhibitor as a potential treatment for SEPN1-RM. Meanwhile, a clinical trial with TUDCA might be worthwhile in patients with SEPN1-RM.

### Limitations of the study

Our study identifies ERO1 as an important mediator of SEPN1-RM pathogenesis and suggests ERO1 as a valuable biomarker of SEPN1-RM. However, more studies on muscle biopsies of patients with SEPN1-RM are necessary to understand whether there is any effect of specific SEPN1 mutations or the muscle type on ERO1 expression. We also suggest genetic inhibition of ERO1 and the chemical chaperone TUDCA as valid treatments for SEPN1-RM. It is still enigmatic why TUDCA has a beneficial effect on the SEPN1-RM since it is not a direct ERO1 inhibitor; quite likely, ERO1, in the absence of a functional SEPN1, triggers proteotoxicity, and TUDCA relieves such a detrimental condition.

## STAR★Methods

### Key resources table

**Table:** 

REAGENT or RESOURCE	SOURCE	IDENTIFIER
**Antibodies**		

Mouse pan-actin (clone C4)	Sigma Aldrich	Cat#MAB1501, RRID:AB_2223041
Mouse GAPDH	Abcam	Cat#AB9484, RRID:AB_307274
Monoclonal mouse anti-FLAG M2	Sigma Aldrich	Cat#F3165, RRID:AB_259529
Monoclonal mouse anti-KDEL	Enzo life Sciences	Cat#ADI-SPA-827, RRID:AB_10618036
Selenoprotein N (A11)	Santa Cruz Biotechnology	Cat#SC-365824, RRID:AB_10844630
Mouse oxPHOS antibody	Abcam	Cat#Ab110413, RRID:AB_2629281
Rabbit EROL	Ester Zito’s laboratory	^49^ Publication
Anti-FLAG M2 affinity gel	Sigma-Aldrich	Cat#F2426, RRID:AB_2616449
Goat anti-mouse IRDye 680RD	LI-COR Biosciences	Cat#926–68070, RRID:AB_10956588
Goat anti-Rabbit IRDye 800CW	LI-COR Biosciences	Cat#926–32211, RRID:AB_621843
Goat anti-Rabbit IgG-Peroxidase antibody	Sigma Aldrich	Cat#A6154, RRID:AB_258284
Goat anti-Mouse IgG-Peroxidase antibody	ThermoFisher	Cat#A16066, RRID:AB_2534739
Myosin heavy chain type I Antibody	Developmental Studies Hybridoma Bank DSHB	Cat#BA-D5
Myosin heavy chain type IIA Antibody	Developmental Studies Hybridoma Bank DSHB	Cat#SC-71
Myosin heavy chain type IIB Antibody	Developmental Studies Hybridoma Bank DSHB	Cat#BF-F3
DyLight™ 405 AffiniPure Goat Anti-Mouse IgG, Fcγ subclass 2b specific	Jackson Immunoresearch	Cat#115-475-207
Alexa Fluor® 488 AffiniPure Goat Anti-Mouse IgG (H + L)	Jackson Immunoresearch	Cat#115-545-003
Alexa Fluor® 594 AffiniPure Goat Anti-Mouse IgM, μ chain specific	Jackson Immunoresearch	Cat#115-585-020

**Biological samples**		

Human primary myoblast cultures from SEPN1-RM patients	Genethon Biobank/Cochin Bank	Table S2
Human muscle biopsies	Genethon Biobank/Cochin Bank	Table S2

**Chemicals, peptides, and recombinant proteins**		

Harris hematoxylin	BIO-OPTICA	Cat#05–06004/L
Eosin Y	BIO-OPTICA	Cat#05–10002/L
Nitrotetrazolium Blue Cloride	Sigma Aldrich	Cat#N6876-250MG
β-Nicotinamide adenine dinucleotide (NADH)	Sigma Aldrich	Cat#N8129
WGA	ThermoFisher	Cat#W11261
WGA, Alexa Fluor 647 conjugate	Thermo Fisher Scientific	Cat#W32466
Direct Red	Sigma Aldrich	Cat#365548
Fluormount	Diagnostic BioSystems	Cat#K024
DPX	Sigma Aldrich	Cat#06522
Protease inhibitors	Roche	Cat#11697498001
DTT(Dithiothreitol)	Sigma Aldrich	Cat#D0632
Thapsigargin	Sigma Aldrich	Cat#T9033
InstantBlue	Expedeon	Cat#ISB1L
NEM	Sigma Aldrich	Cat#E3876
IAA	Sigma Aldrich	Cat#I1149
Trypsin Sequencing Grade	Roche	Cat#11047841001
C18 (Octadecyl) Empore Disk	3M	Cat#98-0604-0217-3
Acetonitrile HPLC LC-MS Grade	VWR	Cat#83640.320
Formic Acid LC-MS Grade	Thermo Scientific	Cat#28905
ReproSil-Pur C18-AQ 1.9 μm beads	Dr. Maisch Gmbh	Cat#R119.aq
SureBeads protein G magnetic beads	Bio-Rad Laboratories	Cat#1614023
Dulbecco’s modified Eagle’s medium (DMEM) low glucose, 110 mg/L sodium pyruvate	Gibco	Cat#10567014
Dulbecco’s modified Eagle’s medium (DMEM) high glucose, 110 mg/L sodium pyruvate	Gibco	Cat#21969-036
Fetal Bovine Serum	Euroclone	Cat#ECS0180L
Penicillin-Streptomycin	EuroClone	Cat#ECB3001D
FURA 2a.m.	Molecular probes, Thermo Scientific	Cat#F1221
MitoTrackerTM Red CMXRos	Invitrogen	Cat#M7512
Formaldehyde 37%	Sigma-Aldrich	Cat#252549
Dulbecco’s Phosphate Buffered Saline PBS	Euroclone	Cat#ECB4004L
EN460	Millipore	Cat#328501
Taurooursodeoxycholic Acid Sodium Salt	EMD Millipore Corp.	Cat#580549
Passive lysis buffer	Promega	Cat#E194A
Type I collagenase	Sigma-Aldrich	Cat#C0130
RNase-Free DNase Set	Qiagen	Cat#79254
RNase Inhibitor	Thermo Fisher	Cat#N8080119
CellTiter 96 AQueous MTS Reagent Powder	Promega	Cat#G1112
Phenazine methosulfate	Sigma-Aldrich	Cat#P9625
Naproxene	Sigma-Aldrich	Cat# PHR1040
Methanol	Carlo Erba	Cat# 412722
Acetic acid	Fluka	Cat# 49199
Ammonium acetate	Sigma-Aldrich	Cat# A1542
Trypsin-EDTA 1X in PBS	Euroclone	ECB3052
HEPES buffered saline solution, 2x	Sigma-Aldrich	51558-50ML
Calcium chloride dehydrate	Sigma-Aldrich	C-5080
Hank’s Balanced Salt Solution - HBSS 1X	Gibco	14025092
Triton™ X-100	PanReac AplliChem	A1388
Gelatin from bovine skin	Sigma-Aldrich	G9382
Mowiol® 40-88	Sigma-Aldrich	81386
Coelenterazine	Santa Cruz Biotechnology	sc-205904
Sodium chloride	Sigma-Aldrich	S9888
Potassium chloride	Sigma-Aldrich	P3911
Potassium phosphate monobasic	Sigma-Aldrich	P0662
Magnesium sulfate heptahydrate	Sigma-Aldrich	63138
Magnesium chloride	Sigma-Aldrich	M8266
HEPES	Sigma-Aldrich	H3375
D-(+)-Glucose	Sigma-Aldrich	G8270
Histamine dihydrochloride	Sigma-Aldrich	H7250
Digitonin	Sigma-Aldrich	D141
Calcium chloride solution	Sigma-Aldrich	21115
D-Luciferin	Duchefa Biochemie	L1349
M.O.M.® (Mouse on Mouse) Blocking Reagent	Vector laboratories	MKB-2213
Ultrapure Laminin, mouse	Corning	354239

**Critical commercial assays**		

BCA assay	Pierce	Cat#23227
High capacity cDNA reverse transcription kit	ThermoFisher	Cat#4368814
RNeasy Plus Mini Kit	Qiagen	Cat#217004
ATP Determination Kit	Invitrogen	Cat#A22066
Mitochondria isolation kit	ThermoFisher	Cat#89801

**Deposited data**		

Mass spectrometry	PRIDE	PXD047157
RNA sequencing data on mouse diaphragms	EMBL-EBI	E-MTAB-12460

**Experimental models: Cell lines**		

HeLa WT, SEPN1 KO, ERO1 KO, DKO	Ester Zito’s laboratory	Publication^3^
C2C12 WT, SEPN1 KD	Ester Zito’s laboratory	Publication^2^
HEK 293T WT, ERO1 KO	Ester Zito’s laboratory	This publication
Human primary myoblast cultures from SEPN1-RM patients	Genethon Biobank/Cochin Bank	Table S2

**Experimental models: Organisms/strains**		

Mouse GM C57BL/6J	Ester Zito’s laboratory	N/A

**Oligonucleotides (RT-qPCR primers)**		

SLC25A20	This paper	F: GTT TGT CTG GAC AGC CAC CTA TG R: AGA AGC ACA CGG CGA ACA TAG G
TIMM10	This paper	F: TGC GTG CCT CCC CAC TAC AAG R: ATC CTC TCA TGG ATG TCC AAG TAC
GPX4	This paper	F: CCT CTG CTG CAA GAG CCT CCC R: CTT ATC CAG GCA GAC CAT GTG
MRPL35	This paper	F: GTC ACA TCT GTT GGA CAC CTG G R: TCT CTT GCC TTT CCG TGT GCT
hs-ERO1	This paper	F: GGC TTC TGG TCA AGG GAC R: TGC TTG CAT GTA GGC CAG ATA

**Recombinant DNA**		

ERO1-Lα HDR Plasmid	Santa Cruz Biotechnology	Cat#SC-401747-HDR
pCDNA3-ERO1L-Myc	Ester Zito’s laboratory	Publication^3^
FLAG-SEPN1^C427S,U428C^	Ester Zito’s laboratory	Publication^2^
FLAG-SEPN1^C427S,U428S^	Ester Zito’s laboratory	Publication^2^
pcDNA3 roGFP2 plasmid	David Ron’s laboratory	Publication^1^
Mitochondrial-targeted luciferase enzyme (mtLUC)	Tito Calì’s laboratory	Publication^50^
SPLICS^ER−MTshort^	Addgene	164108
SPLICS^ER−MTlong^	Addgene	164107

**Software and algorithms**		

NIS elements JOBS	Nikon	https://www.microscope.healthcare.nikon.com/it_EU/products/software/nis-elements
MassLynx version 4.1 software	Waters Corp.	N/A
Gene Ontology (GO) ER and Oxidative stress response gene set (version 3)	Harmonizome database collections	https://maayanlab.cloud/Harmonizome/
Enriched functional analysis	EnrichR	https://maayanlab.cloud/Enrichr/
STRING analysis API (version 12)	STRING consortium database	https://string-db.org/
Mascot 2.6.0	Matrix Science	N/A
Proteome Discoverer 1.4	Thermo Scientific	N/A
Scaffold 4.11.1	Proteome Software Inc., Portland, OR	https://support.proteomesoftware.com/hc/en-us
GraphPad Prism® 9	GraphPad Prism	https://graphpad.com
ImageJ	ImageJ	https://imagej.nih.gov/ij/
“Quantification 1” and “Quantification 2” plugin	Tito Cali’s lab	https://github.com/titocali1/Quantification-Plugins
“VolumeJ” plugin	Tito Cali’s lab	https://github.com/titocali1/Quantification-Plugins
GIMP	Open Source	https://www.gimp.org/
DESeq2 (v.1.36.0)	Publication^51^	https://bioconductor.org/packages/release/bioc/html/DESeq2.html
RStudio (v. 2023.06.0 + 421)	Posit.co	https://posit.co/download/rstudio-desktop/
edgeR (v.3.38.4)	Publication^52^	https://bioconductor.org/packages/release/bioc/html/edgeR.html
Limma (v.3.52.2)	Publication^53^	https://bioconductor.org/packages/release/bioc/html/limma.html
Bash (v. 5.0.17(1)-release (x86_64-pc-linux-gnu) [Ubuntu 20.04.6 LTS])	Bash Ubuntu (Linux)	https://help.ubuntu.com/community/Beginners/BashScripting
bcl2fastq (v. 2.20.0.422)	Illumina	https://support.illumina.com/content/dam/illumina-support/documents/documentation/software_documentation/bcl2fastq/bcl2fastq2-v2-20-software-guide-15051736-03.pdf
STAR (v. 2.7.9a)	Publication^54^	https://github.com/alexdobin/STAR
SMASH – semi-automatic muscle analysis	Smith LR, Barton ER. SMASH – semi-automatic muscle analysis using segmentation of histology: a MATLAB application. Skeletal Muscle. 2014; 4(1):21	https://skeletalmusclejournal.biomedcentral.com/articles/10.1186/2044-5040-4-21
Ionwizard 5.0 software	IonOptix corporation	https://www.ionoptix.com/products/software/ionwizard-core-and-analysis/

**Other**		

Tecan Infinite M200	Tecan	N/A
ChemiDoc MP	ThermoFisher	N/A
Nikon A1 confocal system	Nikon	N/A
Stage Top Incubator with Oko touch control system	Oko-lab	N/A
Leica SP5-TCS-II-RS inverted confocal microscope	Leica Microsystem	N/A
PerkinElmer EnVision multimode plate reader	PerkinElmer	N/A
EASY-nLC 1200	Thermo Fisher Scientific	Cat#LC140
Q Exactive HF	Thermo Fisher Scientific	N/A
Alliance 2695 HPLC Separations Module	Waters Corp	N/A
Micromass Quattro *micro* API tandem quadrupole system	Waters Corp	N/A
Gemini C18 chromatographic column	Phenomenx Inc	Cat# 00B-4435-B0
Leica DM6B	Leica Microsystem	N/A
802B Permeabilized Fiber Test Apparatus	Aurora Scientific	N/A
Myopacer	IonOptix corporation	N/A

### Resource availability

#### Lead contact

Further information and requests regarding the manuscript should be directed to the lead contact Ester Zito (ester.zito@marionegri.it)

#### Materials availability

The authors declare that all results supporting the findings of this study are available within the paper and the Supplementary figures. All reagents generated in this study are available from the Lead Contact with a completed Materials Transfer Agreement.

#### Data and code availability

• This paper does not report the original code
• Proteome data are available via ProteomeXchange with identifier PXD047157.
• The RNA-Sequencing dataset has been submitted to the Annotare Database at EMBL-EBI, accessible via https://www.ebi.ac.uk/fg/annotare/, and can be found under the accession number E-MTAB-12460. The Differential Expression Analysis was based on transcriptome profiling, employing the DeSeq2 package within the RStudio environment.
• Gene Set Enrichment Analysis (GSEA) was executed using data derived from the aforementioned RNA-Sequencing Analysis. Bar plots illustrating the results were generated utilizing the ggplot2 package in RStudio. For the GSEA, Gene Ontology Biological Process (GOBP) categories, specifically the (PERK, IRE1, ATF6)-mediated Unfolded Protein Response Pathways, were obtained from the GSEA-Molecular Signatures Database (MSigDB), available at https://www.gsea-msigdb.org/gsea/msigdb.
• Additional details necessary for the reanalysis of the data presented in this manuscript are available upon request.

### Experimental model and subject details

#### Human primary myoblast cultures from SEPN1-RM patients

All human samples were obtained upon informed consent from the patients or their parents (specifically including consent for research use) approved by local ethics committees. Myoblasts were obtained from diagnostic muscle biopsies or from surgically-discarded tissues obtained during therapeutic procedures (scoliosis corrective surgery) from patients homozygous or compound heterozygous for *SEPN1* mutations by genomic DNA sequencing (SEPN1-RM) and from gender- and age-paired healthy individuals (controls). All samples were established by and sourced from certified biobanks (Genethon Biobank or the Cochin Biobank in France). The underlying *SELENON* mutations were identified for diagnostic purposes by certified diagnostic laboratories (mostly Pitié-Salpêtrière and Grenoble Hospitals, France) and available to us after anonymization.

Myoblast samples from three SEPN1-RM patients carrying null or missense *SEPN1* mutations (P1 to P3, mutations detailed in Table S2) were included in this study. Primary myoblast cultures were further enriched for CD56 surface marker via FACS and were maintained in collagen coated plates and myogenic cell culture medium containing DMEM/F10 (50:50), 20% FBS, 2.5 ng·mL−1 fibroblast growth factor 2 (FGF-2 also known as bFGF) and 1% penicillin–streptomycin.

#### Cell lines

SEPN1 KO, ERO1 KO and DKO HeLa cells were described elsewhere.^1^^,^^3^^,^^23^ HEK 293T cells were transfected with ERO1-Lα CRISPR-Cas9 KO plasmids (SC-401747 for human Santa Cruz Biotechnology). The plasmids were co-transfected with homology-directed repair HDR (SC-401747-HDR for human, Santa Cruz Biotechnology) plasmids, which led to the insertion of puromycin resistance gene and red fluorescent protein (RFP) gene as in.^23^^,^^55^ WT and SEPN1 KD C2C12 were described elsewhere.^2^ Cells were routinely tested for Mycoplasma by PCR.

#### Animals

The ERO1A KO mouse line was resuscitated from the embryos of David Ron’s stock. WT and ERO1 KO (C57BL/6J) mice were bred in our animal facility and genotyped accordingly to a previously described protocol.^56^ SEPN1 KO mice and its cross with CHOP KO mice to obtain double (SEPN1, CHOP KO) KO mice were previously described.^3^ SEPN1 KO mice were crossed with ERO1 KO to obtain the double KO (SEPN1, ERO1 KO). Males were separated from females at weaning. All the functional experiments were done on males. Procedures involving animals and their care were conducted in conformity with ARRIVE 2.0 principles and the following laws, regulations and policies governing the care and use of laboratory animals: Italian Governing Law (D.lgs 26/2014, authorization number 19/2008-A issued 6 March 2008 by Ministry of Health; 485/2018, 774/2019, 764/2019-PRauthorization to E.Zito); Mario Negri Institutional Regulations and Policies providing internal authorization for people conducting animal experiments (Quality Management System Certificate—UNI EN ISO9001: 2008—registration number 6121); the NIH Guide for the Care and Use of Laboratory Animals (2011 edition); EU directives and guidelines (EEC Council Directive 2010/63/UE).

#### TUDCA treatments

TUDCA Sodium Salt by EMD Millipore Corp. (ref. 580549-5GM) was dissolved in sterile water at a concentration of 0.05 mg/μl and then filtered using the 0.22μm syringe filter (Starlab) under the hood. TUDCA was injected intraperitoneally (i.p.) at a dose 0.5 mg/g per day for three weeks in five-month-old SEPN1 KO male mice and in the wild-type counterparts. A counterpart of WT and SEPN1 KO animals were treated only with the vehicle (sterile water). Every seven days the animal were weighed to check for weight loss. During the treatments the mice were routinely inspected and did not show any change in their behavior or signs of distress. Twenty-four hours after the last dose of TUDCA, mice were sacrificed.

### Method details

#### Western blotting

Cells were lysed in cold buffer containing 150 mM NaCl, 20 mM HEPES pH 7.5, 10 mM EDTA and 1% Triton X-100, and supplemented with protease inhibitors cocktail (Roche) and 20 mM NEM. Protein concentration was determined by standard BCA assay (Pierce). Samples with equal protein concentration were mixed with non-reducing Laemmli buffer (62.5 mM Tris-HCl pH 6.8, 2% SDS, 10% glycerol and 0.01% bromophenol blue) and heated for 5 min at 95°C. For reducing SDS-PAGE, samples were supplemented with 100 mM DTT. Protein samples separated by either reducing or non-reducing SDS-PAGE were then transferred to Protran nitrocellulose membrane (Merck) and probed with the following antibodies: mouse pan-actin (clone C4) and mouse GAPDH (AB9484, Abcam) from Sigma Aldrich, monoclonal mouse anti-FLAG M2 (F3165, Sigma Aldrich), monoclonal mouse anti-KDEL (ADI-SPA-827, Enzo life Sciences), Selenoprotein N (A-11) from Santa Cruz Biotechnology, mouse OXPHOS (ab110413) from Abcam and rabbit ERO1.^49^

#### Immunoprecipitation

293T cells were lysed in cold Steven’s lysis buffer supplemented with protease inhibitors cocktail (Roche) and 20 mM NEM. Samples containing 1 mg of total protein were pre-cleared using SureBeads protein G magnetic beads (Bio-Rad Laboratories) for 1 h and incubated with 20 μl of EZview Red anti-FLAG M2 affinity gel (Sigma-Aldrich) for 16 h at 4°C. Beads were then washed 4 times with lysis buffer, and immunoprecipitated proteins were detached from beads by heating to 70°C for 5 min in 2x non-reducing Laemmli buffer.

#### Mass spectrometry

FLAG-immunoprecipitated proteins were resolved on a non-reducing 10% SDS-PAGE gel and stained by Coomassie. A slice around 175 kDa was excised and free thiols cysteines were N-ethylmaleimide alkylated by 55mM NEM; then oxidized thiols were reduced by 10 mM DTT and alkylated by 55 mM IAA; proteins were digested overnight by trypsin. Acidified peptide mixtures were desalted and concentrated on StageTipC18 and injected on a nLC-ESI-MS/MS quadrupole Orbitrap QExactive-HF mass spectrometer (Thermo Fisher Scientific) connected to an Easy-nLC 1200 (Thermo Fisher Scientific) equipped with n-column 75 μm ID, 25 cm length, packed in-house with 1.9 μm beads ReproSil-Pur C18-AQ (Dr. Maisch Gmbh, Ammerbuch, Germany). Chromatographic peptide analysis was performed using a 25 min gradient from 95% solvent A (2% ACN, 0.1% formic acid) to 50% solvent B (80% acetonitrile, 0.1% formic acid), followed by a 5 min washing gradient from 50% to 100%, at a constant flow rate of 250 nL/min, for a total run time of 33 min. MS data were acquired using a DDA top 15 method, and the survey full scan MS spectra (300–1,750 Th) were acquired in the Orbitrap with 60000 resolution, AGC target 1e6, IT 120 m. For HCD spectra, resolution was set to 15,000, AGC target 1e5, IT 120 m; normalized collision energy 28% and isolation width of 3.0 m/z. Proteins were identified processing raw files with Proteome Discoverer (version 1.4, Thermo Fischer Scientific). MS/MS spectra were searched with Mascot engine (version 2.6.0, Matrix Science) against the database uniprot_cp_Human_2020 with the sequences of SEPN1 and ERO1 (accession numbers P11111, P22222 and P33333) and setting the parameters: enzyme: trypsin; maximum missed cleavage: 2; variable modifications: carbamidomethylation (C), oxidation (M), protein N-terminal Acetylation, N-ethylmaleimide (C), and N-ethylmaleimide + water (C); peptide mass tolerance: 10 ppm; MS/MS tolerance: 20 mmu. MS/MS-based peptide and protein identification were validated using Scaffold (version Scaffold_4.11.1, Proteome Software Inc., Portland, OR). Peptide identifications were accepted if they could be established at greater than 95.0% probability. Protein identifications were accepted if they could be established at > 99.0% probability and contained at least 2 identified peptides. Protein probabilities were assigned by the Protein Prophet algorithm. Data are available via ProteomeXchange with identifier PXD047157.

#### Confocal ratiometric microscopy with roGFP2

The pcDNA3 roGFP2 plasmid encoding the ratiometric sensor SS_FLAG_roGFP2 (where SS is an artificial signal sequence) under the control of a CMV promoter was a kind gift from David Ron. RoGFP2- transfected cells were analyzed by confocal ratiometric microscopy on a Nikon A1 confocal scan unit with a 40× objective at 1.49 zoom, managed by NIS elements software. Images at 512x512 pixels were obtained using laser excitation of 405 or 488 nm and emission light collected with a 525/50 nm filter, with a sequential scanning mode to avoid bleed-through effects. Four randomly selected fields per condition were acquired longitudinally and analyzed as follows. We adjusted the laser power and the gain of the microscope based on the WT group at baseline (i.e., before DTT or TG exposure), and applied a correction factor on the *Ratio Plus* plug-in of ImageJ to have baseline values of Ex_405_Em_525_/Ex_488_Em_525_ at ∼1. The quantification was done in the area occupied by the fluorescent cells, which were segmented automatically after background normalization to obtain a mask for quantification. The analysis of all groups was referred to the WT baseline, which was one.

#### Short- and long-range MAMs probes

HeLa cells with the four divergent genotypes and WT and SEPN1 KD (knock down) C2C12 were grown in Dulbecco’s modified Eagle’s medium (DMEM) high glucose, 110 mg/L sodium pyruvate (Gibco), supplemented with 10% (v/v) Fetal Bovine Serum (FBS) (Gibco) and 100 μg/mL Penicillin–Streptomycin (EuroClone), at 37 °C in 5% CO_2_ atmosphere. Twenty hours before transfection, cells were seeded onto 13-mm diameter glass coverslips in a 24-multiwell plate (30 000 cells/well) (for immunofluorescence analyses) or into a 6-multiwell plate (300 000/well) (for ATP measurement). The transfection followed the standard Ca^2+^ phosphate procedure. For one 13-mm diameter coverslip 3 μg of SPLICS^ER−MT^ short or long probes were used, while for one well of the 6-multiwell plate 12 μg of mtLUC were used. The growth medium was replaced with fresh medium right before transfection. After 8 h, cells were washed at least three times with Dulbecco’s Phosphate Buffered Saline (D-PBS) (EuroClone) and fresh DMEM was added.

#### Immunofluorescence analyses of MAMs

Twenty-four hours after transfection, to visualize the mitochondrial network and assess its integrity cells were incubated with MitoTrackerTM Red CMXRos (Thermo Fisher):100nM of MitoTrackerTM Red CMXRos dissolved in HBSS 1X (Gibco) for 20 min (min) at 37°C with 5% CO_2_. Then, cells were fixed with 3.7% formaldehyde (Sigma-Aldrich) in D-PBS for 20 min at room temperature (RT). Cells were then washed three times with D-PBS and permeabilized with a 0.1% Triton X-100 Bio-Chemica solution (PanReac AppliChem) in D-PBS for 20 min at RT. Finally, cells were washed three times in 1% gelatine (Type B from bovine skin; Sigma-Aldrich) solution in D-PBS for 15 min at RT. After three final washes in D-PBS, coverslips were mounted with Mowiol 40–88 (Sigma-Aldrich).

#### Acquiring images and analyses

Coverslips were observed a few hours after mounting, with a Leica SP5 confocal microscope equipped with 63× HCX PL APO objective, and a numerical aperture of 1.40. Images were acquired at laser wavelengths of 488 and 570, every 0.29 nm step on the z axis. Images were analyzed using ImageJ (National Institutes of Health (NIH)) and the SPLICS^ER−MT^ *puncta* were counted as in.^26^

#### Mitochondrial ATP measurements in live cells

Twelve hours after transfection with Mitochondrial-targeted luciferase enzyme (mtLUC), cells were re-plated into a 96-multiwell White/Clear Bottom Plate, TC Surface (Thermo Fisher) (10x10^3^ cells/well) for the luminescence measurements by a PerkinElmer EnVision plate reader equipped with two injector units. For the ATP measurements, cells were washed twice with a modified Krebs Ringer Buffer (KRB: 135mM NaCl (Sigma-Aldrich), 5mM KCl (Sigma-Aldrich), 0.4mM KH2PO4 (Sigma-Aldrich), 1mM MgSO4(7 H2O) (Sigma-Aldrich), 1mM MgCl2 (Sigma-Aldrich), 20mM HEPES (Sigma-Aldrich), pH 7.4), glucose (0.1% D-(+)-Glucose (Sigma-Aldrich))/Ca^2+^ (1mM Calcium chloride solution (Sigma-Aldrich) and placed in 50 μL of KRB + glucose/Ca^2+^. After recording the basal background signal for 5 s (s), a 20 μM (final concentration) D-luciferin (Duchefa Biochemie) solution in KRB + glucose/Ca^2+^ was added and, since luciferin is cell-permeable, the luminescence was detected immediately. After 100 s, when the signal reached a plateau, a 100 μM (final concentration) histamine solution in KRB + glucose/Ca^2+^ with 20 μM (final concentration) D-luciferin was added to boost ATP production. For each measurement, the luminescence signal (CPS, counts per second) after histamine was normalized on the mean CPS at the plateau reached after the first luciferin addition.

#### MTS viability assay

25x10^3^ WT and SEPN1 KD C2C12 cells were grown in low glucose (1,5 g/L) DMEM for 24 h, then treated with 5 microM of EN460, a known ERO1 inhibitor,^27^ or 1mM TUDCA. After further 24 h of treatment the viability and mitochondria activity was assessed through an MTS viability assay. MTS [3-(4,5-dimethylthiazol-2-il)-5-(3-carboxymetoxyiphenil)-2-(4-solfophenil)-2H-tetrazolio] and PMS (Phenazine methosulfate) were added to cells as indicated in the CellTiter 96 Aqueous Non-Radioactive Cell Proliferation Assay (Promega). Acquisitions were made by TECAN infinite M200 using excitation wavelengths at 490 nm

In parallel experiments, cells were grown in 6-cm plates. ER stress/UPR was evaluated by Real-time quantitative RT-PCR of the indicated gene products as in^3^ and ERO1 and BIP by Western Blotting.

#### ATP quantification in muscles

Muscles were collected and frozen in N-pentane and then lysed mechanically using pestle and mortar and liquid nitrogen. Then, ten volumes of Passive Lysis Buffer 1X Ref E194A (Promega, Madison WI USA) were added, and muscles homogenized with Turrax. The samples were centrifuged for 15 min at 4 °C at the maximum speed to remove the insoluble material. The protein concentrations were calculated by BCA assay. For the ATP assay, the muscle lysates were diluted at a concentration of 50 ng/ul to load 500 ng in the assay and ATP measured by ATP Determination Kit by Invitrogen (Thermo Fisher Scientific), Cat. A22066.

#### Mitochondrial protein quantification in muscles

Muscles were collected and weighted. Mitochondria were isolated by Mitochondria Isolation Kit for Tissue (Thermo Fisher Scientific, Cat. 89801) and lysed in RIPA buffer. Mitochondrial protein concentration was determined by standard BCA assay (Pierce).

#### TUDCA levels in diaphragms and plasma

Diaphragms and plasma were collected from vehicle and TUDCA treated mice (WT and SEPN1 KO), and muscle tissue were homogenized in RIPA lysis solution (1:10, w/v). After the addition of the naproxen (internal standard, IS) to the homogenate or plasma, the samples were mixed with 1% acetic acid in cold methanol (1:10, v/v) and centrifuged for protein precipitation. Then, supernatants were dried under nitrogen and the residues were re-suspended and injected into the HPLC-MS/MS system (HPLC Alliance 2695 - Micromass Quattro micro API triple quadrupole, Waters). Separation was done following a gradient elution (mobile phase A, 0.1% CH_3_COOH in ammonium acetate 25mM; mobile phase B, 0.1% CH_3_COOH in MeOH) on a Gemini C18 column (Phenomenex Inc) with and mass spectrometric analysis was done with a triple quadrupole mass spectrometer in negative ion mode and multiple reaction monitoring (MRM) mode, measuring the fragmentation products of the deprotonated pseudo-molecular ions (quantitation ion transitions: TUDCA, m/z 498.4 → m/z 124.1; IS, *m/z* 229.3 → *m/z* 170.0). Diaphragm and plasma samples of treated mice were run and analyzed in parallel with calibration curves linear in the range 0.1–100 μg/g and 0.02–20 μg/mL respectively.

#### RNA sequencing on diaphragms

RNA sequencing was performed on diaphragms of male mice: WT, SEPN1 KO, CHOP KO, SEPN1 KO/CHOP KO (DKO2) old (18 months); WT, SEPN1 KO, CHOP KO, SEPN1 KO/CHOP KO (DKO2) young (2 months); WT, SEPN1 KO, ERO1 KO, SEPN1 KO/ERO1 KO (DKO) young (9 months) (N = 4 for each genotype and age).

RNA was extracted from diaphragms with the Qiagen RNeasy kit and quantified with Nanodrop; quality was measured using Qubit. RNA Sequencing (RNA-Seq) was done on the Illumina NextSeq500 with single-end, 76 base pair long reads. The overall quality of sequencing reads was evaluated using FastQC (v.0.11.9). Sequence alignments of total-RNA (stranded) to the reference mouse genome (GRCm39) were done using STAR (v.2.7.9a) in two-pass mode. Gene expression was quantified at the gene level using the comprehensive annotations made available by Gencode (vM27 GTF File). Samples were adjusted for library size and normalized with the variance stabilizing transformation (vst) in the R statistical environment using DESeq2 (v1.28.1) pipeline. GSEAs were performed using the limma (v.3.44.3) package. Gene-set collections were retrieved from the Molecular Signature Database (MSigDB). p-values were corrected for multiple testing using the false discovery rate (FDR) procedure, with the significance threshold set to 0.05. The raw data are available in the Annotare database EMBL-EBI (https://www.ebi.ac.uk/fg/annotare/) under the accession numbers: E-MTAB-12460.

#### Real-time quantitative RT-PCR analysis

RNA was reverse-transcribed and analyzed using the Applied Biosystems’ real-time PCR System and the ΔΔCt method. Relative gene expression in cells was normalized to GAPDH mRNA levels. The primer sequences of UPR (mouse) transcripts are described in^3^ and the sequences of OXPHOS transcripts and human ERO1 are reported in Key Resources Table.

#### Stainings/histology

Transverse frozen 8-μm cross sections of diaphragm muscles of mice were collected on polylysed slides and stained with hematoxylin and eosin (H&E) solution, Nicotinamide Adenine Dinucleotide Tetrazolium Reductase (NADH-TR), Wheat Germ Agglutinin (WGA) or used for SIRIUS red staining. Minimal Feret’s diameter in muscle fibers was quantified on WGA staining of muscles, that was used to define the fiber perimeter and then, the image was processed by ImageJ software.

#### Hematoxylin and eosin (H&E)

Slides were dried and immersed in filtered Harris’ hematoxylin (ref. 05–06004/L, Bio-Optica Milano) for 4 min. The slides were washed in water and immersed for 1min in Eosin Y 1% (ref. 05–10002/L, Bio-Optica Milano) then washed in water. The slides were dehydrated in ethanol: 10s in 70% ethanol, 20s in 80% ethanol and then two times in 100% ethanol for 30s. Finally, the slides were immersed in xylene (ref. 492301, CARLO ERBA Reagents S. A. S., 616 BP F-27106 Val de Reuil Cedex) for 3 min. DPX was used as a mounting reagent.

#### Nicotinamide Adenine Dinucleotide Tetrazolium Reductase (NADH-TR)

Staining solution was prepared with 25mg of Nitrotetrazolium Blue Cloride (Ref. N-6876, SIGMA-ALDRICH, Co. 3050 Spruce Sreet, St. Louis, MO 63103 USA), 20mg of β-Nicotinamide adenine dinucleotide (NADH) (Ref. N-8129) (SIGMA-ALDRICH, Co. 3050 Spruce Sreet, St. Louis, MO 63103 USA) dissolved in TRIS-HCL 0.1M pH = 7,4. The slides were dipped in the staining solution for 40 min at 37°C. After the slides were washed in: 30% acetone for 3 s, 60% acetone for 3 s, 90% acetone for 3 s, 60% acetone for 3 s and 30% acetone for 3 s. Finally, slides were washed for three times in distilled water, then dried. DPX was using as mounting reagent.

#### Wheat Germ Agglutinin (WGA)

Once the slides are dried, a line was drawn around the muscle slices using a PAP PEN, a liquid repellent slide marker pen. The slices were fixed in Paraformaldehyde 4%, dissolved in PBS, for 5 min at room temperature (ref. 76240 Fluka Chemie GmbH CH-9471 Buchs, Sigma Aldrich Chemie GmbH, Riedstr., D-89555 Steinheim). The slides were permeabilized in Triton 0,1% diluted in PBS, for 5 min at room temperature (Ref. T8787 Sigma Aldrich Co. 3050 Spruce Street, St. Louis, MO 63103 USA) and then, washed in PBS at room temperature for 5min, three times. WGA (Ref. W11261 Molecular Probes, Life Technologies, Eugene, OR, USA) was diluted 1:1000 in PBS and added to the slices overnight. The next morning, slices were washed in PBS at room temperature for 10min, three times. The slides were dried and mounted with Fluormount Ref. K024 (Diagnostic BioSystems, 6616 Owens Drive, Pleasanton, CA 94588/Emergo Europe, Prinsessegracht 20, 2514 AP, The Hague, The Netherlands).

#### Muscle fiber type composition

Thin 10μm cryosections of diaphragm muscles were stained for myosin. To perform the myosin isoform staining, sections were blocked for 1h at RT with mouse-on-mouse (MOM, MKB-2213, Vector laboratories), then incubated with primary antibodies over night at 4°C. The following antibodies were used: types I MyHC (BAD5), IIa MyHC (SC-71) and IIb MyHC (BF-F3). All myosin isoform antibodies have been obtained from the Developmental Studies Hybridoma Bank, created by the NICHD of the NIH and maintained at The University of Iowa, Department of Biology, Iowa City, IA 52242. Secondary antibodies were added for 1h at 37°C (Dylight 594-conjugated anti-mouse IgM Jackson 115-585-020, 488-conjugated anti-mouse IgG Jackson 115-545-003 and 405-conjugated anti-mouse IgG2b 115-475-207) in goat Serum 4% PBS. Wheat germ agglutinin (WGA, Alexa Fluor 647 conjugate, Thermo Fisher Scientific W32466) was used to highlight fibers limits. Picture have been obtained using Leica DM6B fluorescent microscope, and have been analyzed for using a combination of SMASH software (SMASH – semi-automatic muscle analysis using segmentation of histology: a MATLAB application) to obtain the segmentation mask, and ImageJ software to measure fibers cross sectional area and myosin isotype distributions.

#### Sirius red

The slides were fixed with 96% ethanol for 2min, washed with tap water for 10min and then with distilled water for 2min, two times. Later, they were treated with 0,2% aqueous phosphomolybdic acid (Ref. P-7390 Sigma Aldrich, 3050 Spruce Street, St. Louis, MO 63103 USA) for 5min, and then stained with 0,1% Direct Red ref. 365548 (SIGMA-ALDRICH, Co. 3050 Spruce Sreet, St. Louis, MO 63103 USA/SIGMA-ALDRICH CHEMIE GmbH, Riedstr. 2, 89555 Steinheim, Germany), dissolved in a saturated picric acid solution ref. 102362680 (SIGMA-ALDRICH, Co. 3050 Spruce Sreet, St. Louis, MO 63103 USA/SIGMA-ALDRICH CHEMIE GmbH, Riedstr. 2, 89555 Steinheim, Germany), 0.5g in 500mL, for 30min. The slides were washed with 0.01M HCl for 5min 2 times, dehydrated in a graded series of ethanol: 30 s in 70% ethanol, 30 s in 95% ethanol and lastly, two times in 100% ethanol for 1min and 3min. The slides were immersed in Xilene ref. 492301 (CARLO ERBA Reagents S. A. S., 616 BP F-27106 Val de Reuil Cedex) for 2min. Finally, the slides were dried and the cover glass was applied after DPX.

#### Electron microscopy (EM)

Intact diaphragms were fixed at room temperature with 3.5% glutaraldehyde in 0.1M Na Cacodylate buffer, pH 7.2 for several hours. Small pieces of fixed diaphragms were then postfixed in 2% OsO4 in the same buffer for 2 h and then block stained in aqueous saturated uranyl acetate. After dehydration, specimens were embedded in an epoxy resin (Epon 812). Ultrathin sections (∼50 nm) were cut using a Leica Ultracut R microtome (Leica Microsystem) with a Diatome diamond knife (Diatome Ltd.) and double-stained with uranyl acetate and lead citrate. Sections were viewed in an FP 505 Morgagni Series 268D electron microscope (FEI Company), equipped with Megaview III digital camera and Soft Imaging System at 60.

#### Quantitative analyses of EM images

Data contained in Tables were collected from diaphragms muscle fibers (3 mice for each group analyzed). The incidence of both CRUs and mitochondria as well as that of mitochondria-CRUs pairs was determined from electron micrographs of non-overlapping regions randomly collected from longitudinal EM sections by counting their number and reporting the average per area (100 μm^2^) of section. If an individual mitochondrion extended from one I band to another, it was counted in both. In each EM image, we also determined the number of mitochondria at A band and reported as average number per 100 μm^2^.

#### EM sample size

Date resulted from the following sampling: 23 fibers from 18-month-old WT mice, 22 fibers from 18-month-old SEPN1 KO mice; 25 fibers from 6-month-old SEPN11 KO mice; 7 fibers from 6-month-old CHOP KO mouse; 12 fibers from 6-month-old CHOP, SEPN1KO (DKO2) mice; 10 fibers from 6-month-old ERO1, SEPN1 KO (DKO) mice; 10 fibers from 6-month-old TUDCA, SEPN1 KO mice. For each group, 5 micrographs/fiber were analyzed.

#### Calcium handling in FDB

A culture of living skeletal muscle fibers from mouse Flexor Digitorum Brevis (FDB) muscle is obtained by treating the tissue with Tyrode buffer (NaCl 140mM; KCl 2mM; CaCl2 0.5mM; MgCl2 2mM; HEPES 10mM; 5mM glucose), 0,2% type I collagenase (Sigma-Aldrich C0130) and 10% fetal bovine serum (FBS, Sigma Aldrich) for 1h at 4°C. The samples are then incubated for 1h at 36°C, gently mixing the solution every 10 min. The muscles are transferred in a Petri dish and washed three times with warm Tyrode buffer 10% FBS, a 1mm diameter sterile glass pipette is used to gently move the tissue to help fiber dissociation. Isolated fibers are placed on coated microscope coverslip coated with mouse laminin (Corning 354239, 60μg/100μL in sterile water) in Tyrode buffer 10% FBS, 1% penicillin-streptomycin-amphotericin, kept in an incubator with 5% CO_2_ at 36.5°C. To measure calcium transients, fibers are incubated with 5 μM Fura-2/AM (Molecular Probes) in Imaging buffer (NaCl, 125mM; KCl, 5mM; MgSO_4_, 1mM; KH_2_PO_4_, 1mM; glucose 5.5mM; CaCl_2_, 1mM e HEPES, 20mM) with 1% BSA for 30 min at 37°C. The depolarization protocol (twitch) using an electrical stimulator (Myopacer: Ionoptix corporation). The ratio between fluorescence excitations at 340nm and 380nm is measured using a photo multiplicator connected to the microscope recording emission intensities at 500nm. Traces are recorded using Ionwizard 5.0 software and analyzed using Ionwizad software.

#### Single skinned fiber tension in diaphragm

A diaphragm strip is pinned to a silicone support in cold skinning buffer (150mM KPr, 5mM KH2PO4, 5mM MgOAc, 5mM EGTA, 2.9mM NaATP, 0.5mM Sodium Azide, 2mM DTT, protease and phosphatases inhibitors, pH 7.0) at 4°C. After 24h the sample is transferred to storage buffer (same as skinning but in 50% glycerol) for 1h at 4°C and then to −20°C. A single fiber is mechanically isolated from the muscle under the stereomicroscope and an aluminum T-clip is applied to each end. The fiber is mounted on an Aurora Scientific 802B setup, one end on the tensiometer hook for tension measurement, the other end on the motor hook to control fiber length. The fiber is placed in a relaxed buffer and an 8% glutaraldehyde and Toluidine blue solution is used to crosslink each end for a few seconds. The solution is changed to remove excess glutaraldehyde and the fiber length can be adjusted to obtain a sarcomere length of 2.5μm. The setup allows the fast exchange of baths, so the fiber can be placed in relaxing, pre-activating and activating buffers to measure maximal isometric tension. Tension values are normalized for the fiber cross-sectional area.

#### ATF4 in primary myoblasts of SEPN1-RM patients

We measured ATF4 through a fluorescent readout. We transfected an expression plasmid encoding the fusion protein ATF4-mscarlet (a gift from Dr. David Andrews; Addgene#115970) using lipofectamine 3000 reagent (Thermofisher) following the standard protocol. On the 3rd day post-transfection, 1x10^4^ myoblasts were seeded in 96-well plate. The ATF4 response was detected 24h after seeding by measuring the mscarlet fluorescent signal at 561nm_ex_/Em570-530nm_em_ using the Flexstation 3 (Molecular devices). The signal was normalized by the total maximum signal from the transfected cells obtained after incubation with hydrogen peroxide (H_2_O_2_, 100μM) for 16h. We measured the signal using four technical replicates for each condition.

#### ATP assay in primary myoblasts from SEPN1-RM patients

1-4x10^3^ cells were seeded in each 96-well plates and cultured them in myoblast media to subconfuency before treating with increasing TUDCA (0-2mM, Sigma) supplements for 16h. The cultures were then challenged with thapsigargin (0-400nM) for another 4h. Viable cells were determined by incubating the cells for 2h at 37C with a fluorogenic cell-permeant peptide Gly-Phe-7-Amino-4-Trifluoromethylcoumarin peptide (MPBio) at 390nm_ex_/505nm_em_ and this signal was used as the normalizer for the ATP measurement. The ATP level in each culture (in relative luminescent units RLU) was measured using the luciferase kit (Promega) and the Centrol microplate luminometer (Berthold). Four technical replicates were done for each condition and the statistical significance (p < 0.05) of the ATP levels in different treatments and between control and SEPN1-RM was determined using two-way ANOVA with Dunnett post-hoc correction.

#### Bioinformatics analysis on human samples

Public target gene sets associated with endoplasmic reticulum (ER) stress response (GO_0034976) and oxidative stress response (GO_0006979), compiled by Harmonizome online database collections, were obtained for the Venn diagram analysis. The subsequent common gene set were used to query for top GO biological process, rare disease and Manhattan plot analysis based on online EnrichR algorithm, which provides an interface to the Enrichr database hosted at https://maayanlab.cloud/Enrichr/. STRING analysis was performed using the algorithm and STRING database version (https://string-db.org/) with the default parameters and minimum interaction score set at 0.3. The resulting interaction network map was further subjected to k-means clustering to obtain clusters of closely interacting protein groups within the network; this method provides a clearer visualization of the queried protein groups that have an enriched biological and or functional connections indicated by same color bubble and the strength (confidence) of the connection was indicated by the thickness of the connecting solid lines (within cluster) and dotted lines (between clusters). The strength, assessed using Log10(observed/expected) to measure the extent of the enrichment effect, indicates the ratio between the count of queried proteins in the network annotated with a specific term and the expected count of proteins annotated with the same term in a randomly generated network of equivalent size. The p values for enrichment describe the significance of this enrichment, determined through false discovery rate statistics with the Benjamini-Hochberg correction for multiple testing within each category.

#### RNA extraction from SEPN1-RM patient frozen muscle samples

Paravertebral or latissimus dorsi samples were obtained from surgically-discarded muscle fragments during spinal surgery for scoliosis correction and donated for biomedical research after informed consent from the patients or their parents. We analyzed samples from three SEPN1-RM patients (including P3, for whom cultured myoblasts were also studied) and from three sex- and age-paired individuals with idiopathic scoliosis without an underlying muscle disease (controls). The samples were initially snap-frozen in liquid nitrogen or in liquid nitrogen-cooled isopentane and stored at −80°C. For RNA extraction, the samples were first sectioned to approximately 10μm thickness and then pulverized in a small metal container chilled with dry ice. The resulting powder was transferred to a 1.5mL Eppendorf tube and lysed directly with RLT buffer, supplemented with 0.1% (v/v) beta-mercaptoethanol. The samples were further lysed with a 1mL Dounce homogenizer on ice and passed through a 21-gauge needle syringe five times to ensure complete cell disruption. Any unlysed particles were pelleted at 8000*g* for 15 s. The resultant lysate, with a volume of up to 600 μL, was subsequently used for RNA extraction following the protocol supplied with the Qiagen RNeasy Plus Mini Kit.

### Quantification and statistical analysis

Data are the mean ± SEM. The GraphPad Prism program (GraphPad Software, Inc. La Jolla, CA, USA) was used for data processing. Statistical significance was established using the unpaired t-test or Mann-Whitney test for two-group analysis and one-way ANOVA multiple comparison tests for three or more groups. For EM, statistical significance was determined using a one-way ANOVA followed by post-hoc Tukey test for multiple comparisons. One asterisk indicates p < 0.05, two for p < 0.01, three for p < 0.001 and four for p < 0.0001.

## Supplemental information

Document S1. Tables S1–S3 and Figures S1 and S2

Document S2. Article plus supplemental information
