# Supplementary material for: SEPN1-related myopathy depends on the oxidoreductase ERO1A and is druggable with the chemical chaperone TUDCA
Source: Cell Rep Med. 2024 Feb 22;5(3):101439. doi: 10.1016/j.xcrm.2024.101439 (PMC10982971; doi:10.1016/j.xcrm.2024.101439)
Supplement: Document S1. Tables S1–S3 and Figures S1 and S2 [file mmc1.pdf]

Cell Reports Medicine, Volume *5*

Supplemental information

SEPN1-related myopathy depends on the oxidoreductase ERO1A and is druggable with the chemical chaperone TUDCA

Serena Germani, Andrew Tri Van Ho, Alessandro Cherubini, Ersilia Varone, Alexander Chernorudskiy, Giorgia Maria Renna, Stefano Fumagalli, Marco Gobbi, Jacopo Lucchetti, Marco Bolis, Luca Guarrera, Ilaria Craparotta, Giorgia Rastelli, Giorgia Piccoli, Cosimo de Napoli, Leonardo Nogara, Elena Poggio, Marisa Brini, Angela Cattaneo, Angela Bachi, Thomas Simmen, Tito Calì, Susana Quijano-Roy, Simona Boncompagni, Bert Blaauw, Ana Ferreiro, and Ester Zito

Supplementary

Supplementary Table 1 related to Figure 1
Supplementary Table 2 related to Figure 1
Supplementary Figure 1 related to Figure 2
Supplementary Table 3 related to Figure 3
Supplementary Figure 2 related to Figure 3

NAMES	TOTAL					ELEMENTS					
ER/Oxidative stress	9	TRAF2	BAK1	MAP3K5	P4HB	BCL2	THBS1	JUN	PARK2	ERO1A/ERO1L	
ER stress	105	CREB3L1	ATP2A1	EIF2B5	ERN2	ATF6	ATF6B	CREB3L2	UBQLN1	AIFM1	SELENOK
		UBA5	TP53	RNF186	PPP1R15A	TMTC3	TRIB3	PDIA2	UBXN6	SEC16A	TTC23L
		NCK1	RNF185	DNAJB12	GORASP2	FLOT1	BCL2L11	HERPUD1	EIF2S1	PIK3R1	PPP1R15B
		ANKS4B	BBC3	DDRGK1	PDIA3	ATF3	ITPR1	THBS4	SYVN1	UFM1	RASGRF1
		SCAMP5	DDIT3	SEL1L2	DERL1	HSPA5	HYOU1	ERP27	FICD	ERLEC1	RCAN3
		MBTPS1	TMEM117	OS9	CASP4	CEBPB	RASGRF2	PDIA4	BCAP31	ERN1	BRSK2
		ATP2A2	PIK3R2	ATG10	USP19	RNF5	DNAJB9	VCP	TMEM33	MARCHF6	MBTPS2
		GSK3B	BAX	UFL1	ATF4	LINC00675	ALOX15	TNFRSF10B	TMCO1	CDK5RAP3	QRICH1
		EIF2AK3	NIBAN1	CFTR	CXCL8	UFC1	ATP2A3	CHAC1	RHBDD1	ERP44	XBP1
		AGR2	WFS1	SEL1L	TMX1	BAG6	DNAJC10	RNF139	PDIA6	CREB3L3	DAB2IP
		SELENOS	TMEM259	HSP90B1	RNF183	CCDC47					
Oxidative stress	276	MSRB1	CA3	MSRA	ATOX1	STK24	PINK1	PTPRK	EDN1	PPARGC1A	GSR
		NDUFS2	GPX6	CYP11A1	NAPRT	BNIP3	TXNRD2	SFTPC	ANXA1	PDGFRB	HMOX1
		VRK2	PRKRA	KPNA4	LPO	PRDX3	PNKP	PXN	SIRT2	LCK	SOD2
		NDUFA6	PNPT1	UCN	CCS	OSER1	MSRB3	OXSR1	MMP3	ERCC1	ZNF580
		AXL	AQP8	HTRA2	MBL2	NGF	PARK7	NR4A2	CAT	LONP1	KLF2
		AGAP3	MPO	ETV5	CRYGD	VKORC1L1	PRDX6	GPX5	PPP2CB	STK25	RAD52
		GPX7	EGLN1	SCGB1A1	AKT1	STK26	ROMO1	JAK2	SLC23A2	CHUK	STX2
		PDK2	LIAS	SRXN1	SEPP1	KRT1	NDUFA12	RRM2B	CPEB2	SPHK1	PDLIM1
		ADAM9	COL1A1	TXNDC8	KCNC2	GSS	TRPA1	NUDT1	IDH1	PLK3	PTGS2
		PON2	GPX3	DUSP1	OLR1	FABP1	MMP14	MDM2	COQ7	NEIL1	ETFDH
		TAT	MAP2K1	EGFR	NFKB1	SIRT1	OXR1	CYGB	MB	CD38	S100A7
		TPM1	SOD1	MSRB2	SGK2	CYP2E1	STC2	GLRX2	EZH2	TRPM2	PSIP1
		PYCR1	KDM6B	NET1	PLEKHA1	VNN1	SDC1	ERCC8	LCN2	MAPK7	AKR1C3
		SETX	CDK1	TXN	SOD3	PXDNL	NDUFS8	TXNL1	FOS	TXN2	CHRNA4

Supplementary Table 1 related to Figure 1

Oxidative stress	ADPRHL2	CRYAB	TP53INP1	PCGF2	APTX	RHOB	GCLM	UCP3	SNCA	ALDH3B1
	XPA	PRDX5	CD36	VIMP	HAO1	RBM11	FOXO3	PXDN	IPCEF1	PSEN1
	MICB	STAT6	DDIAS	FKBP1B	GPX1	APOD	SLC7A11	ADNP2	ANGPTL7	AREG
	MAPK8	ERCC3	APEX1	PPIF	PAX2	GJB2	PRKD1	STAR	NR4A3	TACR1
	DUOX2	FGF8	TXNRD1	MT3	GAB1	LRRK2	PPARGC1B	EPX	PRDX1	NFE2L1
	TPO	MGMT	AIF1	ETS1	ATP7A	RCAN2	PSMB5	TXNDC2	TRPC6	FER
	TNFAIP3	FOXO1	RELA	TXNIP	PRNP	FOSL1	NDUFB4	ADA	STAT1	GCLC
	HYAL2	STX4	PRKAA1	GSTP1	IL6	MGST1	FXN	APP	CYP1B1	PTPRN
	WRN	GPX8	OGG1	CCR7	TMEM161.	SRC	RGS14	HBB	IL18BP	CDK2
	HYAL1	TOR1A	SLC25A24	HDAC6	UCP2	ATRN	ERCC6	ALS2	PRDX2	DUOX1
	ECT2	MTF1	DGKK	PARP1	DHRS2	KLF4	DPEP1	AQP1	GNAO1	HP
	BTK	ADIPOQ	ERCC2	MPV17	NFE2L2	PDCD10	LIG1	GATM	ABL1	SCARA3
	G6PD	GPX2	PTGS1	GPX4	APOE	BAD	CCL19	DHCR24	CYCS	SELK
	PTK2B	ABCC2	CLN8	PKD2	ZNF277	ARG1				

SEPN1-RM patients

Patient	Gender	Muscle biopsy histopathology	*SEPN1* mutations	Other affected relatives	Muscle sampled/at age	Sample(s) used for this study
P1	F	Multi-minicore congenital myopathy	c.77_84dup, exon 1 //c.1315C>T (p.R439X), exon 10		PV/11 years	Primary cultured myoblasts
P2	M	Congenital muscular dystrophy	c.1406G>A (p.R469Q) homozygous exon 11	One affected brother died at age 8 years	PV/14 years	Primary cultured myoblasts
P3	M	Non-specific changes	c.1405C>T (p.R469W) homozygous exon 11	One affected sister died at age 5 years	PV/16 years	Primary cultured myoblasts, Frozen muscle
P4	F	Congenital muscular dystrophy	c.22_23ins10nt (p.Q8PAGPX) homozygous exon 8	Two affected siblings	Latissimus dorsi/13 years	Frozen muscle
P5	F	Multi-minicore congenital myopathy	c.8_12dup homozygous, exon 1		PV/15 years	Frozen muscle

F= female, M=male, PV=paravertebral muscles

Supplementary Figure 1 related to Figure 2

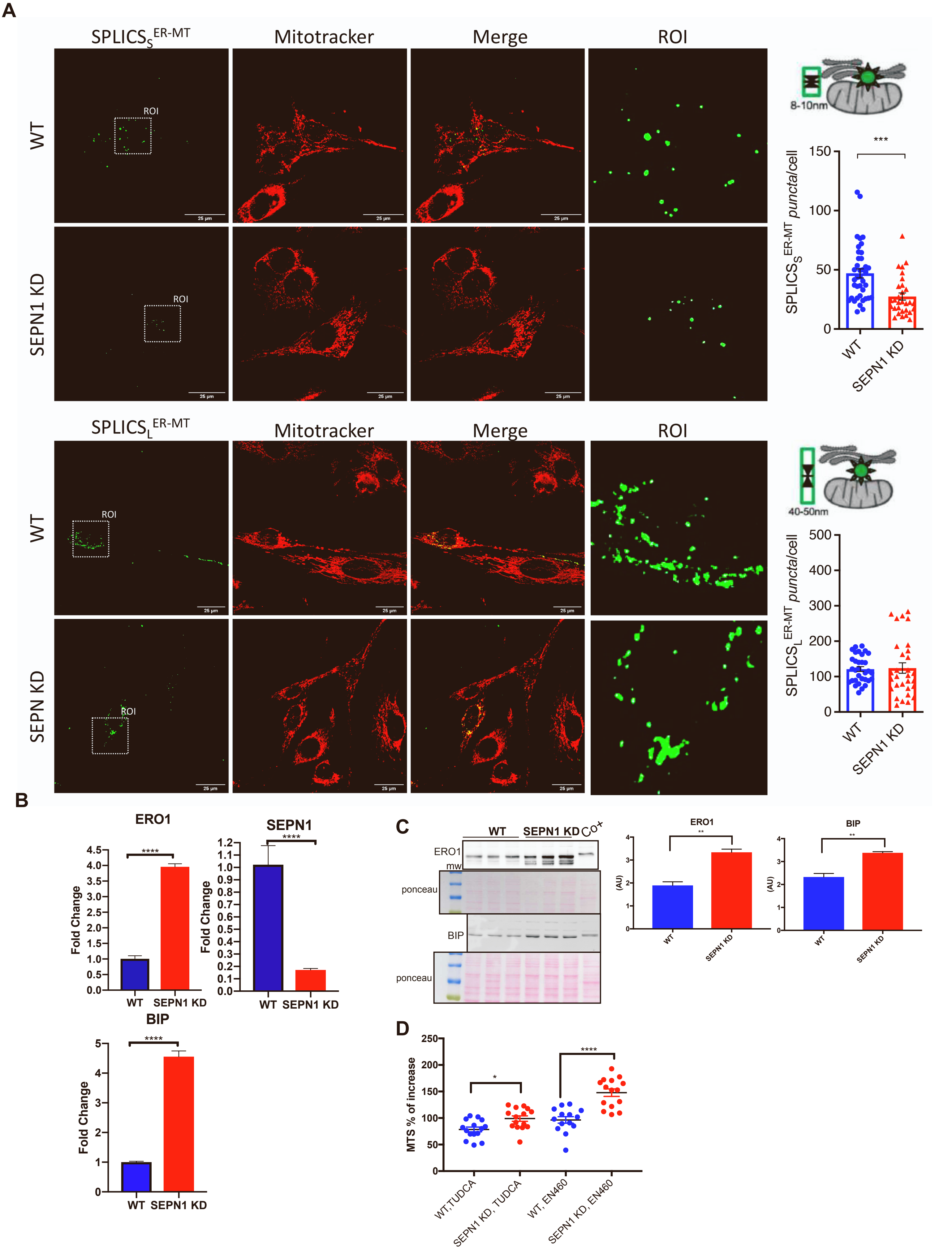

Supplementary Figure 1 related to Figure 2

A Images and quantification of $SPLICS_s^{ER\text{-}MT}$ and $SPLICS_L^{ER\text{-}MT}$ in WT and SEPN1 KD C2C12 cells (N=3, unpaired t-test). **B** Quantitative real-time PCR on cDNA from WT and SEPN1 KD C2C12 cells (N=3, unpaired t-test). **C** Representative Immunoblot from proteins of WT and SEPN1 KD C2C12 cells. Ponceau was used as a loading control. On the right, bar graphs of the quantification in arbitrary unit (AU) (N=3, unpaired t-test). **D** Dot plots showing the percentage of viability in an MTS assay of WT and SEPN1 KD cells treated with EN460 or TUDCA compared to their respective mock-treated cells (N=2, unpaired t-test).

Supplementary Figure 2 related to Figure 3

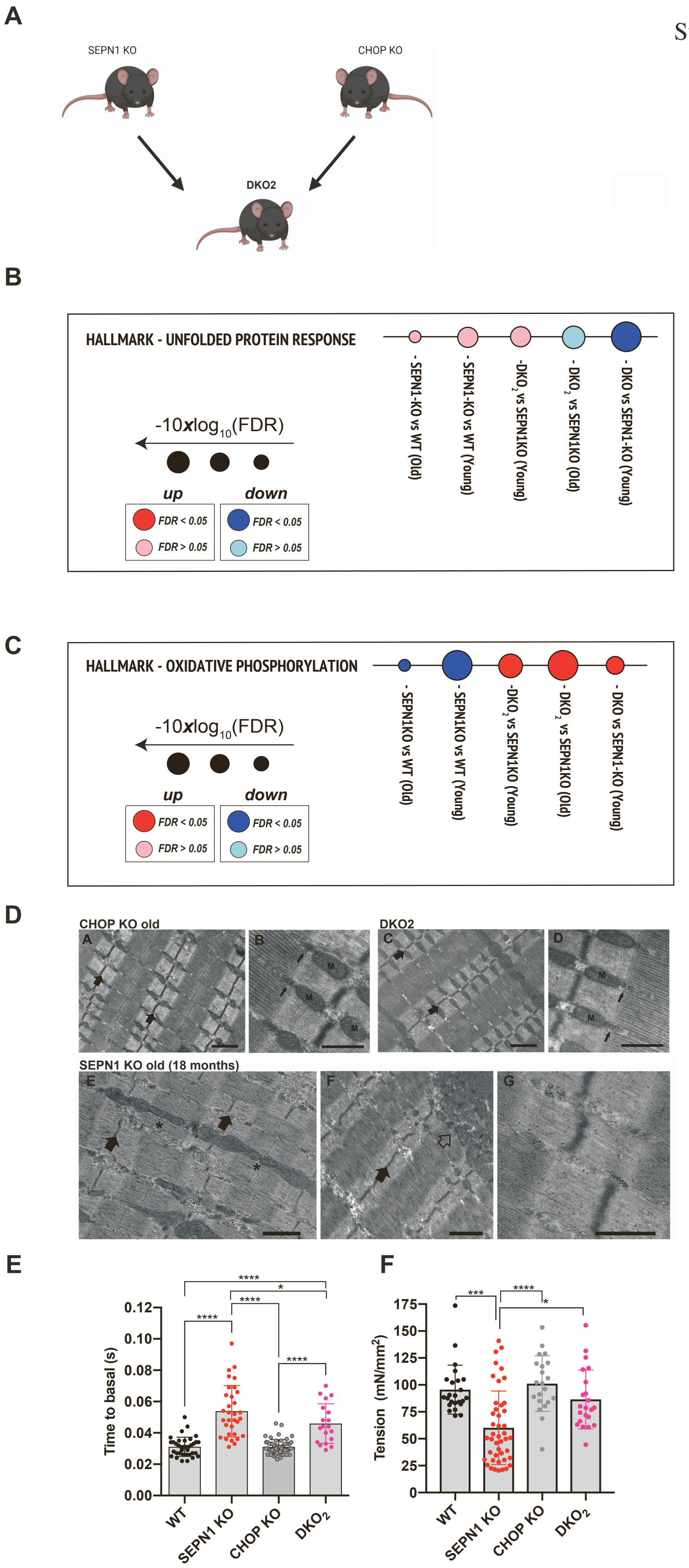

EM quantification and Statistical Analysis.

	No. of CRUs/100μm^2	No. of mitochondria/ 100μm^2	No. of A band mitochondria	No. of CRUs/mitochondria pairs
**WT**	71 ± 4	96 ± 3	3 ± 0	60 ± 3
**SEPN1 KO**	55 ± 3*	71 ± 4*	20 ± 2*	30 ± 3*
**SEPN1 KO (old)**	58 ± 2*	59± 3*#	13 ± 2*#	33 ± 3*
**CHOP KO**	70 ± 3#†	99 ± 3#†	8 ± 1#†	59 ± 2#†
**DKO**	77 ± 3#†	81 ± 3*#†§	9 ± 1#	56 ± 3#†§
**DKO$_2$**	84 ± 2*#†	101 ± 3#†	5 ± 1#†	70 ± 2*#†
**SEPN1 KO, TUDCA**	58 ± 2*§&	73 ± 2 †ǂ§&	6 ± 1#†	50 ± 2*#†§

*Date are shown as mean ± SEM; * p<0.05 vs WT, # p<0.05 vs SEPN1 KO, † p<0.05 vs SEPN1 KO old, ǂ p<0.05 vs CHOP1 KO, § p<0.05 vs DKO2 and & p<0.05 vs DKO. Calcium Release Unit (CRU)*

Supplementary Figure 2 related to Figure 3

A Graphical representation of the cross between SEPN1 KO and CHOP KO mice to get (SEPN1, CHOP KO) DKO2 mice. **B** Dot plots in Hallmark gene sets indicating the up-regulation of UPR in SEPN1 KO and down-regulation in DKO and DKO2 diaphragms of young (2-9 months) or old mice (18 months). **C** Dot plots in Hallmark gene sets indicating the down-regulation of OXPHOS in SEPN1 KO and up-regulation in DKO, DKO2 diaphragms of young (2-9 months) or old (18 months) mice (N=4, each genotype, each age range). **D** Representative EM images of diaphragm from six-month old CHOP KO (panels A and B) and DKO 2 mice (panels C and D) and from SEPN1 KO 18-months-old (old) mice (panels E-F-G). *Labeling:* large arrows point to Z-line; small arrows point to CRUs or triads; asterisk is for longitudinal A-band mitochondria; M is for mitochondria. *Scale bars: A, E, C and G: 1 μ m; B, F, D: 0.5 μ m.* **E** Time to 50% basal measurement of calcium uptake after single pulse stimulation in primary culture of mouse FDB fibers on WT, SEPN1KO, CHOPKO and DKO2 mice as in [1]. **F** Effect of recovery of diaphragm single-fiber tension in DKO2 mice compared to SEPN1KO (N=5 mice/genotype, non-parametric one-way ANOVA, Kruskall-Wallis multiple comparison test).

Reference:

1. Pozzer D*, et al.* A maladaptive ER stress response triggers dysfunction in highly active muscles of mice with SELENON loss. *Redox Biol* **20**, 354-366 (2018).
